# The Active Jasmonate JA-Ile Regulates a Specific Subset of Plant Jasmonate-Mediated Resistance to Herbivores in Nature

**DOI:** 10.3389/fpls.2018.00787

**Published:** 2018-06-14

**Authors:** Meredith C. Schuman, Stefan Meldau, Emmanuel Gaquerel, Celia Diezel, Erica McGale, Sara Greenfield, Ian T. Baldwin

**Affiliations:** ^1^Department of Molecular Ecology, Max Planck Institute for Chemical Ecology, Jena, Germany; ^2^Plant Genetics, Brigham Young University, Provo, UT, United States

**Keywords:** jasmonate signaling, jasmonoyl isoleucine (JA-Ile), direct and indirect defense, plant metabolomics, plant-herbivore interactions, *Nicotiana attenuata*, *Manduca sexta*

## Abstract

The jasmonate hormones are essential regulators of plant defense against herbivores and include several dozen derivatives of the oxylipin jasmonic acid (JA). Among these, the conjugate jasmonoyl isoleucine (JA-Ile) has been shown to interact directly with the jasmonate co-receptor complex to regulate responses to jasmonate signaling. However, functional studies indicate that some aspects of jasmonate-mediated defense are not regulated by JA-Ile. Thus, it is not clear whether JA-Ile is best characterized as the master jasmonate regulator of defense, or if it regulates more specific aspects. We investigated possible functions of JA-Ile in anti-herbivore resistance of the wild tobacco *Nicotiana attenuata*, a model system for plant-herbivore interactions. We first analyzed the soluble and volatile secondary metabolomes of ir*JAR4*xir*JAR6*, as*LOX3*, and WT plants, as well as an RNAi line targeting the jasmonate co-receptor CORONATINE INSENSITIVE 1 (ir*COI1*), following a standardized herbivory treatment. ir*JAR4*xir*JAR6* were the most similar to WT plants, having a ca. 60% overlap in differentially regulated metabolites with either asLOX3 or ir*COI1*. In contrast, while at least 25 volatiles differed between ir*COI1* or as*LOX3* and WT plants, there were few or no differences in herbivore-induced volatile emission between ir*JAR4*xir*JAR6* and WT plants, in glasshouse- or field-collected samples. We then measured the susceptibility of jasmonate-deficient vs. JA-Ile-deficient plants in nature, in comparison to wild-type (WT) controls, and found that JA-Ile-deficient plants (ir*JAR4*xir*JAR6*) are much better defended even than a mildly jasmonate-deficient line (as*LOX3*). The differences among lines could be attributed to differences in damage from specific herbivores, which appeared to prefer either one or the other jasmonate-deficient phenotype. We further investigated the elicitation of one herbivore-induced volatile known to be jasmonate-regulated and to mediate resistance to herbivores: (*E*)-α-bergamotene. We found that JA was a more potent elicitor of (*E*)-α-bergamotene emission than was JA-Ile, and when treated with JA, ir*JAR4*xir*JAR6* plants emitted 20- to 40-fold as much (*E*)-α-bergamotene than WT. We conclude that JA-Ile regulates specific aspects of herbivore resistance in *N. attenuata*. This specificity may allow plants flexibility in their responses to herbivores and in managing trade-offs between resistance, vs. growth and reproduction, over the course of ontogeny.

## Introduction

Plants employ sophisticated defense systems in response to herbivore attack. These include both direct defenses: traits which directly impair herbivore performance such as toxins, antifeedants, and repellents; and indirect defenses, which attract parasitoids or predators of attacking herbivores (Dicke and Baldwin, 2010; Mithöfer and Boland, 2012). Herbivory-induced direct and indirect defenses are regulated by complex signaling systems, activated when plants detect tissue damage and herbivore-derived chemical cues (Erb et al., 2012; Schuman and Baldwin, 2016). A central pathway regulating plant defense metabolites is the jasmonate signaling cascade comprising jasmonic acid (JA) and its derivatives, collectively referred to as jasmonates, and the molecular players in jasmonate biosynthesis, perception, and signal transduction (Erb et al., 2012; Wasternack and Strnad, 2016). JA biosynthesis begins with the hydrolysis of 9,12,15-octadecatrienoic acid (18:3) fatty acids from membrane lipids in the chloroplast. These fatty acids are then oxygenated by 13-lipoxygenases (LOX) to 13*S*-hydroperoxy-18:3 or−16:3 fatty acid peroxides, and then further oxidized and cyclized, leading to the formation of (9*S*,13*S*)-12-oxophytodeinoic acid (OPDA). OPDA is transported to the peroxisome, where several steps of beta-oxidation result in the formation of (3*R*,7*S*)-JA (Wasternack and Hause, 2013). The jasmonates comprise dozens of JA metabolites (Wasternack, 2007; Wasternack and Hause, 2013), including the isoleucine conjugate jasmonyl-isoleucine (JA-Ile) which has been identified as the active jasmonate hormone. The conjugation of JA to Ile is catalyzed by JASMONATE RESISTANT 1 (JAR1) in *Arabidopsis thaliana* and its homologs in other plant species, including JAR4 in *Solanum nigrum* and two enzymes, JARs 4 and 6, in *Nicotiana attenuata* (Staswick and Tiryaki, 2004; Wang et al., 2007; VanDoorn et al., 2011a,b). JA-Ile has been demonstrated in molecular interaction studies to interact more strongly than other jasmonates with their receptor complex (Chini et al., 2007; Thines et al., 2007). Specifically, the isomer (+)-7-iso-JA-L-Ile is perceived by a complex of one or more JAZ (JASMONATE ZIM DOMAIN protein) transcriptional repressor protein(s), inositol pentakisphosphate (InsP5), and the F-box protein COI1 (CORONATINE-INSENSITIVE 1), which is part of a Skp/Cullin/F-box complex (SCF^COI1^) that functions as a ubiquitin ligase (Xu et al., 2002; Chini et al., 2007; Fonseca et al., 2009; Sheard et al., 2010). The binding of JA-Ile to the SCF^COI1^-JAZ-InsP5 complex triggers the ubiquitination and degradation of the JAZ repressor(s) (Chini et al., 2007; Thines et al., 2007; Katsir et al., 2008). Intact JAZ proteins bind to transcription factors which regulate multiple jasmonate-inducible genes involved e.g. in secondary metabolite biosynthesis. JA-Ile-induced JAZ degradation releases these transcription factors, permitting the activation of defense metabolite biosynthesis (De Geyter et al., 2012).

A variety of jasmonates has been shown to exert biological activity in plants (Erb and Glauser, 2010). Furthermore, the JA precursor OPDA, which is an abundant molecule esterified to galactolipids in chloroplasts of *A. thaliana*, also induces COI1-dependent and -independent transcriptional regulation (Stintzi et al., 2001; Taki, 2005; Ribot et al., 2008), changes in intracellular calcium levels (Walter et al., 2007), and alterations of cellular redox status (Böttcher and Pollmann, 2009). OPDA is released by lipase activity present in oral secretions of feeding insects and regulates herbivory-induced transcriptional responses in *A. thaliana* (Schäfer et al., 2011). *cis*-Jasmone is a volatile jasmonate reported to activate defense responses in various plants, including *A. thaliana* and *Triticum aestivum* (Birkett et al., 2000; Bruce et al., 2008). Another volatile, methyl jasmonate (MeJA), elicits a constitutive defense response when over-produced in *A. thaliana* (Seo et al., 2001). However, in *N. attenuata*, herbivore resistance is reduced when MeJA production is upregulated, demonstrating the importance of analyzing biological functions of different jasmonates in diverse plant species (Stitz et al., 2011a). Other JA derivatives, which are thought to be inactive, may be involved in “switching off” jasmonate signaling: 12-O-β-D-glucopyranosyljasmonic acid (12-O-Glc-JA) and 12-OH-JA are abundant metabolites in many plant species, including *Solanum tuberosum, A. thaliana*, and *N. tabacum* (Yoshihara et al., 1989; Helder et al., 1993; Swiatek et al., 2004; Miersch et al., 2008). Interestingly, 12-O-Glc-JA, but not JA or JA-Ile, was shown to activate leaf closure in *Samanea saman* (Nakamura et al., 2011), supporting the idea that biological activities of jasmonates can be species- and tissue-specific.

Applying JA and JA-Ile to plants, as well as genetically manipulating jasmonate and JA-Ile biosynthesis and perception, has revealed that the responses elicited by these two jasmonates only partially overlap. In *Phaseolus lunatus*, for example, JA and JA-Ile treatments differentially regulate light-dependent extrafloral nectar production, an indirect defense which attracts predatory ants (Radhika et al., 2010). In *A. thaliana*, the emission of some herbivory-induced volatiles, such as terpenoids and methyl salicylate, but not green leaf volatiles (GLVs), is dependent on plants' ability to produce JA (Snoeren et al., 2009). Herbivore-attacked *jar1-1* mutants of *A. thaliana* are as attractive as WT plants for the parasitoid *Cotesia rubecula*, indicating that JA-Ile-mediated signaling may not be involved in indirect defense in this plant (Van Poecke and Dicke, 2003), although an analysis of herbivory-induced volatiles in *jar1-1* mutants of *A. thaliana*, to our knowledge, has not yet been reported. Staswick and colleagues also found that JA-Ile is not required for full COI1-mediated resistance to pathogens and does not regulate all COI1-dependent transcriptional responses (Staswick et al., 1998; Suza and Staswick, 2008). Recently, Vandoorn and colleagues demonstrated that in *S. nigrum*, defense responses and resistance to herbivores in the field require jasmonate biosynthesis, and perception via COI1, but not JAR4, suggesting that JA-Ile plays minor roles in herbivore resistance in this plant (VanDoorn et al., 2011a). As of yet, there has not been an integrative analysis of the role of JA and JA-Ile in herbivory-induced direct and indirect resistance. Ideally, such an analysis would be performed in a model system in which herbivory-induced defense metabolites and their role in resistance to natural herbivores have been well characterized.

*Nicotiana attenuata* is an ecological model plant in which defensive roles of many metabolites and herbivore-induced plant volatiles have been demonstrated in nature. This plant specifically responds to attack from a variety of natural herbivores. For example, feeding by the specialist lepidopteran herbivore *Manduca sexta* is perceived via fatty acid-amino acid conjugates (FACs) present in the insect's oral secretions (Halitschke et al., 2003; Bonaventure et al., 2011) and more recently, the plant-derived elicitor 2-hydroxylinolenic acid (2-HOT) was shown to promote the production of resistance metabolites (Gaquerel et al., 2009, 2012). Applying *M. sexta* oral secretions (OS) to wounded leaves of *N. attenuata* dramatically amplifies transient wound-induced JA and JA-Ile production between 20 and 90 min post-elicitation, and the abundance of precursors within 5 min (Schittko et al., 2000; Kallenbach et al., 2010). Silencing JARs 4 and 6 in *N. attenuata* revealed that JA-Ile is not likely to be the only active oxylipin signal regulating direct defense metabolites: plants silenced in JAR4 and JAR6 by RNAi (inverted repeat, ir*JAR4*xir*JAR6*) were deficient in the resistance-related metabolites nicotine and trypsin protease inhibitors (TPI), but produced higher levels of both than did RNAi lines silenced in LIPOXYGENASE 3 (antisense, as*LOX3*), the lipoxygenase providing 18:3 fatty acid hydroperoxides for JA biosynthesis in this plant; ir*JAR4*xir*JAR6* plants were also intermediate between as*LOX3* and WT in their resistance to *M. sexta* (Halitschke et al., 2003; Wang et al., 2008). Microarray analysis of ir*JAR4*xir*JAR6* and as*LOX3* plants supported the conclusion that JA and JA-Ile have partially overlapping but distinct activities (Wang et al., 2008). Importantly, although JAR4 and JAR6 may also regulate conjugation of JA-Leu, which cannot be analytically distinguished from JA-Ile via standard mass spectrometry (MS) analysis, Wang and colleagues showed that JA-Ile application to ir*JAR4*xir*JAR6* plants was sufficient to restore gene expression (except for JAR4 and JAR6), nicotine and TPI production, and resistance to *M. sexta* larvae, to WT levels.

Meanwhile, much more is known about the herbivore-induced metabolome of *N. attenuata*, providing an appealing system in which to systematically investigated herbivore resistance-related metabolomic changes regulated by JA-Ile in comparison to total jasmonates, or jasmonate perception via COI1, and test the importance of JA-Ile vs. total jasmonate biosynthesis for resistance to plants' native herbivores in the field. We compared herbivore resistance of jasmonate-deficient (as*LOX3*) and JA-Ile-deficient (ir*JAR4*xir*JAR6*) plants in a field study, and our data reveal that JA-Ile has specific, and different, effects on herbivore resistance in comparison to jasmonate biosynthesis. Using targeted and untargeted metabolomics, and additional transformed lines manipulating jasmonate signaling (ir*COI1*) and accumulation (s*JMT*), we demonstrate that a large proportion of herbivory-induced changes to the soluble metabolome (known and putative metabolites of direct resistance) are regulated by JA-Ile, but that the biosynthesis of jasmonate-regulated herbivory-induced volatiles, known or putative indirect defenses, is JA-Ile independent. Thus, our study indicates that plants use different jasmonate metabolites to regulate jasmonate-dependent direct vs. indirect resistance.

## Materials and methods

### Plant material

The genotype of *N. attenuata* used in this study was derived from the Desert Inn accession, UT (Baldwin et al., 1994) and wild-type (WT) plants were from the 30th inbred generation. The inverted repeat *JASMONATE RESISTANT 4* (ir*JAR4*) line A-05-355-6 and ir*JAR6* line A-05-380-6 and their hemizygous cross (third transformed generation, T3) were previously described by Wang and colleagues in comparison to independently transformed lines bearing the same construct (Wang et al., 2007, 2008). Two different hemizygous crosses generated from two different independently transformed lines per construct both accumulated only about 16% as much JA-Ile, but the same amount of JA as WT plants after herbivore elicitation; *JAR4* and *JAR6* transcripts were almost undetectable in Northern blots, in comparison to a strong signal for WT plants (Wang et al., 2008). Because possible maternal effects had not previously been tested for, reciprocal hand-pollinated crosses were generated from the ir*JAR4* and ir*JAR6* lines. In most cases a bulk collection of the reciprocal crosses was used for experiments, and where indicated in the Results and figure captions, collections from individual crossings were used. The JAR enzymes conjugate isoleucine to jasmonic acid to generate JA-Ile, but also form some other JA-AA conjugates including JA-Leu, which cannot be analytically separated from JA-Ile by standard LC-MS/MS/MS analysis (Wang et al., 2007). However, Wang and colleagues showed that differences in the transcript accumulation of defense- and growth-related genes between ir*JAR4*xir*JAR6* and WT plants could be restored by the application of pure JA-Ile (Wang et al., 2008). Thus, off-target effects are unlikely.

The antisense (as) *LOX3* line A-300-1 and the ir*COI1* line A-04-249-A-1 were used in the T3 generation, and s*JMT* lines ectopically expressing *Arabidopsis thaliana* jasmonate methyltransferase to channel jasmonate production to MeJA were used in the T2 generation (35S-*jmt*, lines A-07-287-3 and A-07-289-7); all lines were previously described in comparison to independently transformed lines bearing the same construct (Halitschke and Baldwin, 2003; Paschold et al., 2007; Stitz et al., 2011b).

We used WT plants as the control for all experiments. By the second (T2) or third transformed generation (T3) there are rarely measurable effects of the transformation process; screening to avoid off-target insertion effects is part of the normal screening process, and has been done for all lines used here. Consistently, empty vector control plants in only the second transformed generation (T2) have been shown to be indistinguishable from WT plants in their growth and herbivore-induced transcript, hormone and metabolite production (Schwachtje et al., 2008).

### Glasshouse growth conditions

Seed germination and plant growth in the glasshouse were as previously described (Krügel et al., 2002; Adam et al., 2017). Briefly, seeds were germinated on Gamborg B5 medium and kept under 16 h light/8 h dark at 26 °C; 10 d later, seedlings were transferred to small pots (TEKU JJP 3050 104 pots, Poeppelmann GmbH & Co. KG, Lohne, Germany) in the glasshouse and then to 1 L pots 10 d later with soil, fertilization and watering regimes as previously described and grown under 19–35°C, 16 h light (supplemental lighting by Philips Sun-T Agro 400 W and 600 W sodium lights) and 55% humidity.

### Field growth conditions

Importation and release of transgenic plants were carried out under Animal and Plant Health Inspection Service (APHIS) import permit numbers 07-341-101n and release permit number 11-350-101r. Seed germination and seedling growth was as previously described (McGale et al., 2018). Briefly, seedlings were germinated on Gamborg B5 media under illumination from fluorescent lights (GE Plant & Aquarium 40 W and GE Warm White 18 W) at ambient temperatures at the field station. Two to three weeks after germination, seedlings with four visible leaves were transferred into previously hydrated 50-mm peat pellets (Jiffy 703, www.jiffypot.com) treated with Borax to provide boron, an essential micronutrient (1:100 dilution of a 1.1 g/l stock solution) and adapted over 2 weeks to the field conditions of high light intensity and low relative humidity by keeping seedlings first in shaded, closed translucent plastic 34-quart boxes (Sterilite), then opening the boxes, and subsequently transferring open boxes to partial sunlight in mesh tents (Tatonka). Adapted size-matched seedlings were transplanted into an irrigated field plot at the Lytle Ranch Preserve, Santa Clara, Utah, in April 2012.

### Leaf treatments

For glasshouse experiments, the transition leaf, or the second fully expanded leaf (positions 0 or +2) on rosette-stage or elongated plants were used for treatments as described for specific analyses except for the analysis of s*JMT* lines and the accompanying WT, for which the second rosette leaf on elongated plants was used (older than +2, mature and non-senescent). Plants in both the rosette and the elongation stage show robust jasmonate-mediated responses in the glasshouse (Diezel et al., 2011) but it is easier to collect volatiles on-plant from leaves on elongated plants.

To mimic herbivory, leaves were treated by wounding with 3 rows of holes to the lamina on each side of the midvein (6 rows in total) using a tracing wheel, and the addition of 20 μL *M. sexta* oral secretions (W+OS) diluted 1:5 in distilled water, a procedure which has been shown to elicit most responses induced by *M. sexta* herbivory (Halitschke et al., 2001; Schittko et al., 2001). OS were collected from larvae from an in-house colony at the Max Planck Institute for Chemical Ecology fed on WT *N. attenuata* plants.

For elicitation with different jasmonates, purified substances synthesized in-house (JA, JA-Ile, JA-Leu) or obtained from Sigma-Aldrich (*cis*-jasmone) were first checked for purity by liquid chromatography-mass spectrometry analysis (UHPLC-ESI/TOFMS in negative mode, Bruker), and then 0.25 μmol of the pure compound was dissolved per 20 μL of 30% ethanol by first dissolving the corresponding mass in ethanol (Sigma-Aldrich) and then slowly adding distilled water to prevent precipitation; 30% ethanol in distilled water was used as a solvent control. Twenty microliter of jasmonate in 30% ethanol was added to 6 rows of wounds made with a tracing wheel, as described for W+OS treatment. For plants in the field, similar, fully expanded, non-senescent, and minimally damaged leaves were used for control and W+OS-treated samples.

### Analysis of jasmonates

The leaf at the +2 position on glasshouse-grown rosette-stage plants was treated with W+JA and treated leaves were harvested 1, 3, and 6 h later; harvests were conducted as described for the analysis of leaf secondary metabolites. For plants in the field, a similar, fully-expanded, minimally damaged leaf was treated with W+OS and harvested 1 h later and a similar, untreated leaf was harvested simultaneously from control plants into aluminum foil, then frozen immediately on dry ice and kept frozen on dry ice in a −20°C freezer until transport on dry ice to the Max Planck Institute for Chemical Ecology, Jena. Tissue was ground over liquid nitrogen to a find powder and kept at −80°C until extraction and analysis. Jasmonates were measured in ethyl acetate extracts of leaves, re-suspended in 70% methanol, on a Varian 1200L LC-MS/MS/MS system as previously described (Wang et al., 2007; Stitz et al., 2011b). For JA-treated leaves, JA was excluded from measurements. Results were calculated as concentrations in ng mg FM^−1^ using isotopically labeled internal standards except for OPDA and MeJA, for which no internal standard was available; the JA-Ile internal standard was used for all JA conjugates.

### Analysis of leaf secondary metabolites

The transition leaf (position 0) on rosette-stage plants was treated with W+OS and excised at the petiole 72 h later by which time treated leaves had grown to position +2 or +3. Midveins were excised, and leaf tissue was flash-frozen in liquid nitrogen. Tissue was ground over liquid nitrogen to a find powder and kept at −80°C until extraction and analysis. Extracts of leaves in acidified 40% methanol (soluble metabolome) were analyzed on a Bruker UHPLC-ESI/TOFMS in positive ionization mode as previously described (Gaquerel et al., 2010). Chromatograms were exported as netCDF files and peak detection, picking and integration was performed using the R package XCMS (Smith et al., 2006; Tautenhahn et al., 2008), and then ion traces were deconvoluted and putative in-source pseudo-spectra reconstructed with the R package CAMERA (Kuhl et al., 2012) as previously described (Gaquerel et al., 2010; Stitz et al., 2011a). The data matrix was imported to Microsoft Excel and isotopic peaks and multi-charged m/z signals detected by CAMERA were excluded to reduce the redundancy within the data matrix. Consistent mass features—present (for a single plant genotype) in four out of the five biological replicates—with a retention time >50 s were considered for further statistical analysis.

### Leaf headspace analysis from glasshouse-grown plants

The leaf at the +2 position was used, which emits greater amounts of W+OS-induced volatiles than younger or older leaves (Halitschke et al., 2000) except for the analysis of sJMT lines and the accompanying WT, in which a slightly older rosette leaf on elongated plants was used (second rosette leaf, mature and non-senescent). Volatiles were sampled from elongated, pre-flowering plants unless otherwise noted. (Elongation provides easier access to the +2 leaf for volatile collection.) Several hours to 1 day after treatment according to peak emission times of different volatiles, as described in the Results and figure captions, treated leaves were enclosed in two 50 mL PET cups (Huhtamaki, Finland) lined on the edges with foam to protect leaves and with an activated charcoal filter attached to one side for incoming air, and secured with miniature claw-style hair clips as described previously (Schuman et al., 2009). Headspace volatiles were collected for several hours (see section Results and figure captions) on 20 mg of PoropakQ (Tholl et al., 2006) (Sigma-Aldrich) in self-packed filters (bodies and materials from ARS Inc.) by drawing ambient air through these clip cages at 300 mL min^−1^ using a manifold with screw-close valves set to provide equal outflow, via pushing air at 2–3 bar through a Venturi aspirator as previously described (Oh et al., 2012). Background VOCs present in ambient air were collected using empty foam-lined PET cups which were the same as those used for leaves, and background signals were later subtracted if necessary from raw intensities of plant samples prior to further processing. After trapping, Porapak Q filters were stored at −20°C until extraction, which was done by addition of 320 ng of tetralin as an internal standard (IS), and elution of volatiles with 250 μL of dichloromethane (Sigma–Aldrich). Filters were eluted into a GC vial containing a 250 μL glass insert.

For volatile metabolomic analysis, an Agilent 6890N gas chromatograph equipped with an Agilent 7683 autoinjector coupled with a LECO Pegasus III time-of-flight mass spectrometer with a 4D thermal modulator upgrade was used to collect three-dimensional GCxGC-TOFMS data and to generate a peak table as previously described (Gaquerel et al., 2009). The data matrix was imported to Microsoft Excel and consistent mass features—present (for a single plant genotype) in four out of the five biological replicates—with a retention time >150 s were considered for further statistical analysis.

For the targeted analysis of (*E*)-α-bergamotene, samples were analyzed on a on a Varian CP-3800 GC coupled to a Varian Saturn 4000 ion-trap mass spectrometer with a Varian CP-8400 autoinjector equipped with a Phenomenex ZB5 column from (Torrance, CA; 30 m × 0.25 mm i.d., 0.25 μm film thickness) and compounds were separated by a temperature ramp of 5°C min^−1^ between 40 and 180°C as previously described (Oh et al., 2012).

The identification of compounds was conducted by comparing GC retention times and mass spectra to those of standards and mass spectra databases: Wiley version 6 and NIST (National Institute of Standards and Technology) spectral libraries. The (*E*)-α-bergamotene peak was identified by retention index (RI) on two different columns and similarity to spectral libraries, and confirmed using an authentic standard as previously reported (Schuman et al., 2009).

Relative quantification of individual volatile compound peaks was done using the combined peak area of two specific and abundant ion traces per compound using MS Work Station Data Analysis software (Varian) or the ChromaToF software (LECO) and normalized by the 104 + 132 ion trace peak area from tetralin in each sample. The area of trapped leaves was quantified for comparison by scanning and calculating areas in pixels using SigmaScan (Systat Software Inc., San Jose, CA), and subsequently converting pixels to cm^2^ using a size standard which was scanned with leaves. As leaf areas did not differ between lines (Datasheet 1), relative abundance of volatile compounds was expressed as a percentage of the IS peak area. The sesquiterpene β-caryophyllene was used as a standard to confirm that measured peak areas of (*E*)-α-bergamotene were within the linear range of detection.

### Estimation of damage from native herbivores in the field

Observations of herbivore damage to the canopy of elongated plants were made on May 16th, 2012, by visual estimation after training for herbivore damage-type recognition. Damage was calculated as described in Schuman et al. (2012) by identifying damage from specific herbivores according to their characteristic feeding patterns, counting the number of leaves per plant (small leaves were counted as 1/5–1/2 of a leaf based on leaf area and large leaves were counted as 1 leaf), estimating the total percentage of leaf area damage due to each herbivore, and dividing the total leaf area damage from each herbivore by the total number of leaves, a protocol which has been used over 10 years of field studies in this system (e.g., Kessler et al., 2004; Steppuhn et al., 2004, 2008; Meldau et al., 2009; Kallenbach et al., 2012; Schuman et al., 2012). Damage estimates were made by C. D.

### Leaf headspace analysis from field-grown plants

Similar, fully expanded, non-senescent, and minimally damaged leaves on flowering plants were used for control or W+OS-treated samples on May 28th and May 29th, 2012. Flowering plants were used in the field, but not in the glasshouse, as this stage is more likely to receive oviposition from moths of *M. sexta*, which is a pollinator as well as an herbivore (Zhou et al., 2017); but in glasshouse-grown plants, jasmonate-mediated responses, although not the emission of (*E*)-α-bergamotene, are attenuated after flowering (Diezel et al., 2011; Schuman et al., 2014). Thus, we also measured jasmonates from WT plants in the field before and after flowering.

Silicone tubings (STs) were chosen as a more convenient method of field sampling (Kallenbach et al., 2014). ST preparation, volatile sampling from leaves, and TD-GC-QMS analysis on a Shimadzu TD−20 thermal desorption unit connected to a quadrupole GC–MS-QP2010Ultra and equipped with a Phenomenex ZB5 column were conducted as previously described (Kallenbach et al., 2014, 2015). STs were exposed to leaf headspaces for 24 h immediately after W+OS treatment and simultaneously from control plants, with 3 technical replicates per leaf of which one was analyzed and the other two were kept as back-up.

ST samples were stored in tightly sealed amber glass screw-cap 1.5 ml vials at −20°C freezer prior to analysis, and were at room temperatures only during sample transport from Utah, USA to Jena, Germany (< 1 day), which is known not to influence the analysis (Kallenbach et al., 2014, 2015). Identification of volatiles by spectral libraries, relative retention and comparison to standard compounds, and relative quantification of a single abundant m/z trace per peak using the Shimadzu software, as well as background correction based on samples of ambient air, were done as previously described (Kallenbach et al., 2014).

### Statistical analyses

Replicates were only excluded from statistical analyses when there was a valid biological reason to do so (death of plant, loss of leaf due to damage). We started with replicate numbers of 4-5 plants for jasmonate and soluble metabolite analysis in glasshouse experiments, 5–10 plants for volatile analyses in glasshouse experiments, and 10–21 plants for all analyses from the field experiment, and final replicate numbers are reported in figure captions and table legends.

Herbivore damage data and (*E*)-α-bergamotene emission from different plant genotypes were analyzed in SPSS 17.0, or in R 3.2.5 and RStudio 1.0.136 (*cis*-jasmone experiment). Datasets were evaluated for homogeneity of variance and normality using Levene's test and visual inspection (SPSS) or visual inspection of residual and Q-Q plots (R) and when these requirements could not be met after log transformation, nonparametric Kruskal-Wallis tests were used for multiple comparisons and Mann-Whitney *U*-Tests for binary comparisons. Holm-Bonferroni corrections were used to maintain Type I error below α = 0.05 when multiple tests were conducted on the same data, and the adjusted *P*-values are reported.

For soluble and volatile metabolome analysis, statistical evaluation was conducted using the Multiple Experiment Viewer (MEV). Analytes were considered to differ significantly between wild-type and transgenic lines which had a fold-change of 1.5 (up-regulation) or 0.67 (down-regulation) at a significance level of α = 0.05, as determined by Student's *t*-tests corrected for the testing of multiple analytes using the Benjamini and Hochberg false discovery rate (FDR) method.

Corrected integrated peak areas from field-collected headspace samples (Kallenbach et al., 2014), and corrected (visual check of integration) normalized peak areas from phytohormone analysis after W+OS, W+EtOH or W+JA treatment, were analyzed using Metaboanalyst (www.metaboanalyst.ca) (Xia et al., 2009, 2015; Xia and Wishart, 2016). Analytes having zero values for all samples in a treatment group were removed and the remaining peak areas were log-transformed and mean-centered to meet assumptions of normality and homoscedasticity. For headspace samples, after an initial exploratory analysis to visualize differences, data were evaluated by one-way analysis of variance (ANOVA) for each treatment group and Tukey's Honestly Significant Difference (HSD) *post-hoc* tests were performed for analytes with FDR < 0.05, to control for the testing of multiple analytes. Pearson's correlation tables were also calculated and are available as part of the source data files for this manuscript along with the input data and R script (Datasheet 1). For phytohormones, data were evaluated by one- or two-way analysis of variance (ANOVA) and *P*-values were adjusted to an FDR < 0.05, to control for the testing of multiple analytes.

## Results

### JA-Ile regulates ca. 60% of jasmonate-responsive secondary metabolites, but not volatile metabolites

We first asked what portion of the jasmonate-regulated secondary metabolome was likely to be regulated by JA-Ile, as opposed to total jasmonate products of LOX3 activity, or jasmonate signaling mediated by COI1. We investigated both the soluble and volatile secondary metabolome, which include many small molecule metabolites that have been shown to confer direct resistance to herbivores as a result of toxic, antifeedant, or deterrent effects, as well as indirect resistance as a result of increased predation rates on herbivores (Kessler and Baldwin, 2001; Kallenbach et al., 2012; Schuman and Baldwin, 2016). We employed a controlled simulated herbivory treatment comprising wounding and the addition of *M. sexta* oral secretions (W+OS) (Halitschke et al., 2001; Schittko et al., 2001) in order to elicit standardized responses in as*LOX3*, ir*JAR4*xir*JAR6*, ir*COI1*, and WT plants. We then conducted an untargeted analysis of metabolite extracts from tissue samples, and headspace samples: without identifying specific metabolites, we analyzed the patterns in relative abundance of ions (m/z features) after filtering raw mass spectral data to identify ions which likely represented different metabolites (i.e., one ion per metabolite).

**Figure 1:**
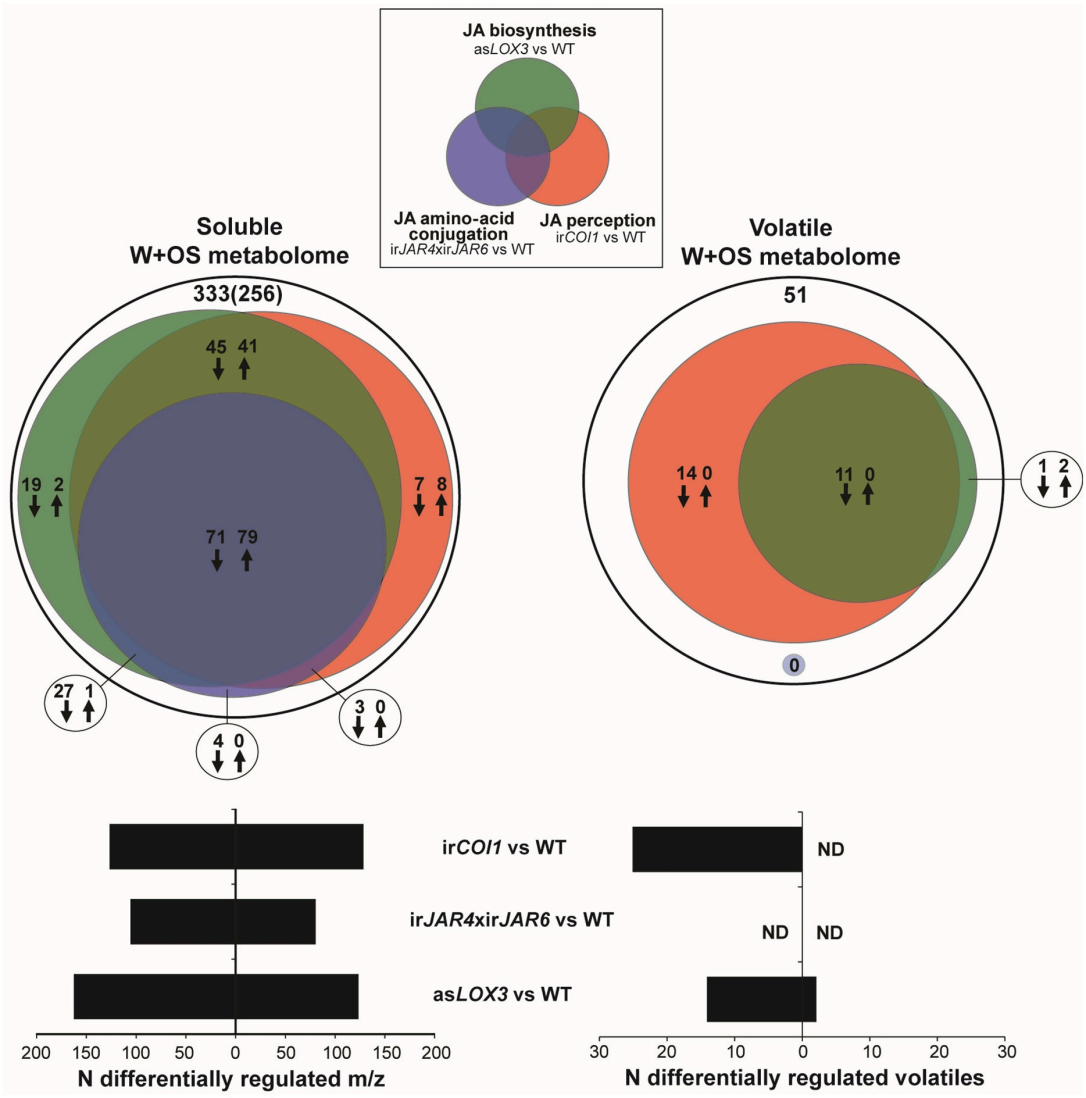
JA-Ile formation regulates a subset of the jasmonate-dependent soluble metabolome, but not volatile emissions induced after simulated herbivore attack. Venn diagram representations of the relative contributions of total jasmonate biosynthesis (as*LOX3* vs. WT), of JA-Ile signaling (ir*JAR4*xir*JAR6* vs. WT), and jasmonate perception (ir*COI1* vs. WT) to metabolic changes activated in *Nicotiana attenuata* rosette-stage leaves during simulated herbivore attack (W+OS) underscore the minor role of JA-Ile formation for induced volatile production. Methanolic extracts (soluble metabolome) of leaves collected 72 h after W+OS (*n* = 5 plants) were analyzed using UHPLC-ESI/TOFMS in positive ionization and processed mass-to-charge ratio (m/z) peak matrices analyzed for differential expression between transgenic lines and WT (Gaquerel et al., 2010). Plant volatiles (volatile metabolome) were collected 0–6 and 6–24 h after elicitation by W+OS (*n* = 5 plants) and analyzed using GCxGC-TOFMS (Gaquerel et al., 2009). Processed volatile matrices obtained for the two collection times were combined and analyzed for differential expression between transgenic lines and WT. Deconvoluted volatiles and m/z features were called deregulated in transgenic lines compared to in WT when their fold change (ratio of averages: transgenic line vs. WT) was higher than 1.5 (up-regulation: up arrows) or lower than 0.67 (down-regulation: down arrows) at a significance level of 0.05 (*t*-test, *P* < 0.05 corrected for multiple testing using the Benjamini and Hochberg false discovery rate method). Black circles depict the total number of W+OS-regulated features in WT leaves. For the soluble metabolome, the number of predicted metabolites after informatic-deconvolution (see section Materials and Methods) and clustered metabolite-derived m/z in-source fragments is presented in brackets.

We found that JA-Ile synthesis controlled by JAR4 and JAR6 was required for ca. 60% of the response of the soluble secondary metabolome to W+OS treatment, as estimated by the difference between WT and ir*COI1* or as*LOX3* plants, in comparison to the difference between WT and ir*JAR4*xir*JAR6* plants (Figure 1, Supplementary Table 2). In contrast, the abrogation of JA-Ile synthesis in ir*JAR4*xir*JAR6* plants had no significant effect at all on the jasmonate-dependent emission of at least 25 herbivore-induced volatiles (Figure 1).

Measurements of peak herbivore-induced JA and JA-Ile accumulation from the same plants used for metabolite sampling showed that asLOX3 plants were deficient in JA (15% of WT values) and JA-Ile (29% of WT), while ir*JAR4*xir*JAR6* were deficient in JA-Ile (20% of WT) but not JA (99% of WT), and ir*COI1* produced less JA (21% of WT) but similar JA-Ile levels (106% of WT) (Table 1, Supplementary Figure 1). In addition, we analyzed known JA-Ile metabolites and abscisic acid (ABA) in these samples. Overall, in addition to the significant differences in JA and JA-Ile, we also found differences in OH-JA-Ile and COOH-JA-Ile, although the levels of these JA-Ile metabolites were low at 1 h after elicitation; and only weak differences in ABA (Table 1).

**Table 1:** ABA, JA, and JA-Ile levels and JA-Ile metabolites in lines deficient in JA-Ile synthesis (ir*JAR4*xir*JAR6*), total jasmonate biosynthesis (as*LOX3*), or jasmonate perception (ir*COI1*) vs. wild-type (WT) plants during peak JA and JA-Ile accumulation, 1 h after W+OS treatment (*n* = 3–5 plants).

**Analyte**	***F*_(3, 17)_^a^**	**Adj. *P***	**Tukey^b^**	**Genotype**	**Mean ± SE (ng g^−1^ FM)^c^**
JA	67.28	<**0.0001**	**a**	WT	3,833 ± 354
			**a**	ir*JAR4*xir*JAR6*	3,799 ± 413
			**b**	as*LOX3*	585.4 ± 91.3
			**b**	ir*COI1*	820.1 ± 92.0
JA-Ile/Leu	38.35	<**0.0001**	**a**	WT	244.6 ± 28.0
			**b**	ir*JAR4*xir*JAR6*	49.10 ± 3.82
			**b**	as*LOX3*	71.13 ± 12.03
			**a**	ir*COI1*	259.5 ± 26.0
OH-JA-Ile	10.07	**0.0009**	**a**	WT	124.2 ± 14.8
			**ab**	ir*JAR4*xir*JAR6*	30.29 ± 6.19
			**a**	as*LOX3*	40.40 ± 12.91
			**b**	ir*COI1*	10.26 ± 3.78
ABA	3.652	**0.0391**	ns^d^	WT	260.2 ± 9.3
			ns	ir*JAR4*xir*JAR6*	283.0 ± 22.5
			ns	as*LOX3*	203.9 ± 33.7
			ns	ir*COI1*	105.7 ± 9.7
COOH-JA-Ile	ns	ns	ns	WT	8.265 ± 1.280
			ns	ir*JAR4*xir*JAR6*	7.332 ± 2.417
			ns	as*LOX3*	5.277 ± 2.138
			ns	ir*COI1*	4.722 ± 0.9662

*One-way ANOVAs were conducted to identify significant differences (false discovery rate-adjusted P-value, Adj. P < 0.05) after log transformation and mean-centering to achieve normality and homogeneity of variance. Significant differences are indicated in bold. See also Supplementary Figure 1 and Figure 3B*.

a *Degrees of freedom*.

b *Significantly different contrasts (P < 0.05) in Tukey post-hoc tests*.

c *Calculated based on closest internal standard: JA, ABA, or JA-Ile (used for JA-Ile/Leu, OH-JA-Ile, COOH-JA-Ile)*.

d *ns, not significant*.

### Jasmonoyl isoleucine (JA-Ile) deficiency accounted for ca. 30% of jasmonate-mediated resistance to herbivores in nature

We then askedwhether the active jasmonate hormone JA-Ile is responsible for jasmonate-mediated resistance to herbivores for *N. attenuata* plants in nature. We evaluated the herbivore resistance of ir*JAR4*xir*JAR6* plants, deficient in JA-Ile biosynthesis, in comparison to as*LOX3* plants deficient in the synthesis of all jasmonates, or wild-type (WT) controls, by estimating the total canopy damage to field-grown plants by native herbivores. We chose to use asLOX3 and not irCOI1 because asLOX3 plants had a similar level of JA as JA-Ile deficiency, and the reduction in both hormones was similar to the reduction of JA-Ile in ir*JAR4*xir*JAR6* compared to WT plants, in contrast to the more complex changes in jasmonate biosynthesis in ir*COI1* plants (Table 1, Paschold et al., 2008). In this experiment, we did not aim to compare resistance due to jasmonate perception by COI1, but only relative resistance due to JA-Ile production vs. the production of all jasmonates. Because jasmonate-inducible defense in leaves is reported to be strongest prior to flowering (Diezel et al., 2011), we monitored plant canopy damage inflicted by herbivores before plants flowered.

**Figure 2:**
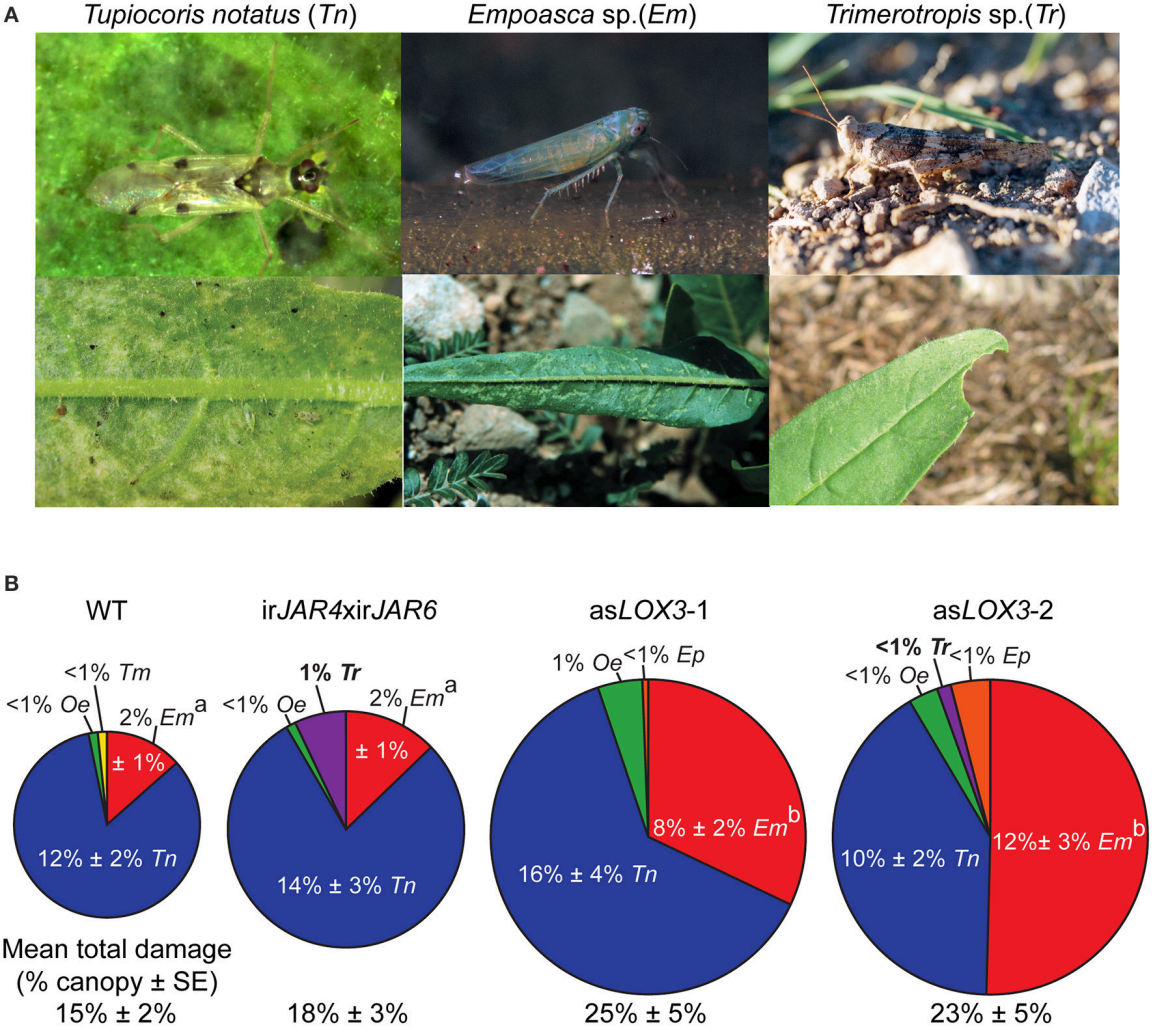
Plants deficient in the active jasmonate JA-Ile (ir*JAR4*xir*JAR6*) were only marginally more susceptible to native herbivores compared to as*LOX3* plants deficient in all jasmonate hormones. **(A)** Upper row: pictures of herbivores which caused more than 1% canopy damage on at least one of the three *Nicotiana attenuata* genotypes investigated (*Tupiocoris notatus, Tn*; *Empoasca* spp., *Em*) or which caused significantly different levels of damage across genotypes (*Empoasca* spp., *Em*; *Trimerotropis* sp., *Tr*). Lower row: damage typical of each herbivore. Photographs C. Bruetting (*Tn* adult), A. Kessler (*Em* and *Em* damage, reproduced with permission from Kessler et al., 2004), A. Steppuhn (*Tr* adult), and D. Kessler (*Tn* damage, *Tr* damage). **(B)** Plants deficient in JA-Ile (ir*JAR4*xir*JAR6*) suffered only 20% more damage from native herbivores than wild-type (WT) plants (mean percent canopy damage ± SE), while plants deficient in total jasmonate biosynthesis (as*LOX3*) suffered 65% more damage. *Trimerotropis* sp. (***Tr***) caused at most 1% canopy damage, but only to ir*JAR4*xir*JAR6* or as*LOX3* plants. In contrast, *Empoasca* spp. (*Em*), opportunistic herbivores sensitive to plant jasmonate signaling capacity, caused more damage on as*LOX3* plants than on either WT or ir*JAR4*xir*JAR6*. The labels as*LOX3*-1 and as*LOX3*-2 refer to a pair of plants of the same transformed line placed at two different positions within each experimental quadruplet (*n* = 20–21); similarities between these two groups indicate the reproducibility of the data set. ^a, b^Different letters indicate significantly different levels of *Empoasca* spp. damage (*P* < 0.05 after a Holm-Bonferroni *post-hoc* correction) in Mann-Whitney *U*-tests following a significant Kruskal-Wallis test across all genotypes.

We found that plants deficient only in JA-Ile suffered 20% more herbivore damage than WT, while as*LOX3* plants, deficient in total jasmonate production, suffered 66% more herbivore damage than WT plants (Figure 2). When comparing damage from specific herbivores, ir*JAR4*xir*JAR6* plants, deficient in JA-Ile, were more susceptible only to *Trimerotropis* spp. grasshoppers, which did not damage WT plants, but caused at most 1% of the total canopy damage in this year. The as*LOX3* plants, deficient in total jasmonates, were also attacked by *Trimerotropis* spp., and were furthermore significantly more damaged by *Empoasca* sp. leafhoppers than were either WT or ir*JAR4*xir*JAR6* plants (Figure 2 and figure caption).

These data indicate that JA-Ile regulates specific aspects of jasmonate-mediated herbivore resistance in *N. attenuata*.

### JA-Ile does not regulate herbivore-induced volatile emissions in field-grown, flowering plants

The metabolomic profiling experiment shown in Figure 1 was conducted before plants flowered because in the glasshouse, flowering plants have abrogated jasmonate responses, which may affect the induction of soluble secondary metabolites more than volatiles (Diezel et al., 2011; Schuman et al., 2014); and we aimed for a rigorous comparison of these two groups of secondary metabolites.

However, flowering plants are more likely to experience oviposition by the herbivore/pollinator *M. sexta*, and this is the stage for which the importance of volatile-mediated defense is best understood (Kessler and Baldwin, 2001; Schuman et al., 2012; Zhou et al., 2017; Joo et al., 2018). We therefore sampled volatiles from flowering plants as part of the field experiment, in order to determine whether JA-Ile might be important for herbivore-induced volatile emissions at this critical stage. The analysis of these samples was consistent with the results of the glasshouse experiment: as*LOX3* plants had abrogated emission of 9 herbivore-induced volatiles quantifiable in these samples, while ir*JAR4*xir*JAR6* plants had reduced emission of only 1 volatile and emitted even greater amounts of the herbivore-induced sesquiterpene 5-epi-aristolochene in comparison to WT plants (Table 2, Supplementary Figures 2, 3, Supplementary Tables 2, 3).

**Table 2:** Herbivore-induced volatile emission was abrogated in field-grown plants with impaired jasmonate biosynthesis (as*LOX3*), but not JA-Ile biosynthesis specifically (ir*JAR4*xir*JAR6*) (*n* = 5–10 plants).

**Treatment**	**df^a^**	**Analyte**	***F***	**Adj. *P***	**Tukey^b^**	**Genotype**
Control	2, 27	α-Terpineol^c^	25.52	<**0.0001**	**a**	WT
					**a**	ir*JAR4*xir*JAR6*
					**b**	as*LOX3*
		(*Z*)-3-Hexenol^d^	12.84	**0.0022**	**a**	WT
					**a**	ir*JAR4*xir*JAR6*
					**b**	as*LOX3*
		(*Z*)-3-Hexenyl-2-methylbutanoate^d^	11.00	**0.0039**	**a**	WT
					**a**	ir*JAR4*xir*JAR6*
					**b**	as*LOX3*
		**(*****Z*****)-3-Hexenyl isobutanoate**^d^	9.606	**0.0064**	**a**	WT
					**b**	ir*JAR4*xir*JAR6*
					**b**	as*LOX3*
		Unidentified green leaf volatile	6.563	**0.0342**	**a**	WT
					ab	ir*JAR4*xir*JAR6*
					**b**	as*LOX3*
W+OS	2, 20	(*E*)-α-Bergamotene^d^	24.66	**0.0002**	**a**	WT
					**a**	ir*JAR4*xir*JAR6*
					**b**	as*LOX3*
		(*Z*)-3-Hexenol^d^	22.42	**0.0002**	**a**	WT
					**a**	ir*JAR4*xir*JAR6*
					**b**	as*LOX3*
		Unidentified sesquiterpene (RT27.547)	11.63	**0.0064**	ab	WT
					**a**	ir*JAR4*xir*JAR6*
					**b**	as*LOX3*
		α-Terpineol^c^	10.50	**0.0081**	**a**	WT
					**a**	ir*JAR4*xir*JAR6*
					**b**	as*LOX3*
		(*Z*)-3-Hexenyl isobutanoate^d^	9.065	**0.0112**	**a**	WT
					**a**	ir*JAR4*xir*JAR6*
					**b**	as*LOX3*
		(*Z*)-3-Hexenyl-2-methylbutanoate^d^	9.010	**0.0112**	**a**	WT
					**a**	ir*JAR4*xir*JAR6*
					**b**	as*LOX3*
		(*Z*)-3-Hexenyl butanoate^d^	6.396	**0.0341**	**a**	WT
					**a**	ir*JAR4*xir*JAR6*
					**b**	as*LOX3*
		**5-epi-Aristolochene**^d^	6.345	**0.0341**	**a**	WT
					**b**	ir*JAR4*xir*JAR6*
					ab	as*LOX3*
		(*Z*)-3-Hexenyl acetate^d^	6.278	**0.0341**	**a**	WT
					**a**	ir*JAR4*xir*JAR6*
					**b**	as*LOX3*

*One-way ANOVAs were conducted to identify significant differences (false discovery rate-adjusted P-value, Adj. P < 0.05) in the leaf headspace abundance of 49 peaks, of which 36 were above the limit of quantification in the headspace of untreated leaves (control), vs. 38 in headspace samples from herbivore-elicited leaves (wounding plus Manduca sexta oral secretions, W+OS). In total, 5 analytes differed significantly by genotype in control samples (box plots in Supplementary Figure 2) vs. 9 after W+OS treatment (box plots in Supplementary Figure 3). In 12 of 14 cases, the analyte is reduced in asLOX3 plants compared to both WT and irJAR4xirJAR6 plants. In only 2 cases do irJAR4xirJAR6 and WT plants differ (in bold): 1 case in which irJAR4xirJAR6 plants, like asLOX3 plants, emit less [the GLV (Z)-3-hexenyl isobutanoate], and 1 case in which they emit more (the sesquiterpene 5-epi-aristolochene). Indicators of significant differences (P-values and letters) are also in bold*.

a *Degrees of freedom*.

b *Significantly different contrasts (P < 0.05) in Tukey post-hoc tests*.

c *Tentative identification based on relative retention and comparison to spectral libraries*.

d *Identity confirmed using a standard*.

### Jasmonate accumulation is reduced, but herbivore inducibility of jasmonates is increased after flowering in field-grown plants

In order to determine whether jasmonate responses were abrogated by the transition to flowering in field-grown plants, we measured jasmonates in response to simulated herbivory both prior to flowering, when herbivore damage rates were assessed (Figure 2 and above), and after flowering, when volatiles were sampled (Table 2, Supplementary Figures 2, 3, Supplementary Tables 2, 3). Induced levels of JA were attenuated after flowering by ca. 70%, but the fold change between control and induced levels was only 4-fold before flowering and 60-fold after flowering; similarly, induced levels of JA-Ile were reduced by 30% after flowering, while the fold change between control and induced plants was 7-fold before flowering and 140-fold after flowering (Table 3). Timepoint, treatment, and the timepoint^*^treatment interaction were all highly significant in a general linear model on log-transformed data (df: 1, 37; all *P*-values < 0.001; treatment, JA: *F* = 110.5, JA-Ile: *F* = 181.9; timepoint, JA: *F* = 87.78, JA-Ile: *F* = 34.88; interaction, JA: *F* = 32.04, JA-Ile: *F* = 17.60).

**Table 3:** Herbivore-induced jasmonic acid (JA) and jasmonoyl-isoleucine (JA-Ile) levels in WT leaves 1 h after W+OS treatment vs. concurrently harvested, untreated leaves was measured in field-grown plants before and after flowering (*n* = 9–10 plants).

**Timepoint^a^**	**Treatment^a^**	**Analyte^a^**	**Concentration^a^ (ng/g FM)**	**FC^b^**
Pre-flowering	Control	JA	279.1 ± 52.6	4.45
		JA-Ile	25.86 ± 7.40	6.61
	W+OS	JA	1323 ± 230	
		JA-Ile	170.9 ± 32.0	
Post-flowering	Control	JA	6.934 ± 4.632	60.8
		JA-Ile	0.8573 ± 0.3559	139
	W+OS	JA	421.6 ± 77.6	
		JA-Ile	119.2 ± 20.5	

Timepoint, treatment, and the timepoint^*^treatment interaction were all highly significant in a general linear model (see section Results).

a *Mean ± SE*.

b *Fold change W+OS/control*.

These data indicate that JA-Ile is required to elicit a subset of jasmonate-regulated herbivore-induced secondary metabolites, but not herbivore-induced volatiles in *N. attenuata*. Furthermore, although JA and JA-Ile levels may be reduced in the leaves of flowering plants, we found that both remained strongly inducible, and thus we infer that induced jasmonates could maintain the induction of herbivore-induced volatile emission in flowering plants (e.g., Schuman et al., 2014).

### JA-Ile does not elicit the volatile (*E*)-α-bergamotene, a highly effective component of plant jasmonate-mediated resistance to herbivores

Because there were very few changes in the herbivore-induced volatile emission of ir*JAR4*xir*JAR6* plants in comparison to as*LOX3* and ir*COI1* plants in both glasshouse and field experiments and over two growth stages (Figure 1, Table 2), we investigated whether JA-Ile or other known jasmonates elicit volatile emission to mediate herbivore resistance. We used the volatile (*E*)-α-bergamotene (initially reported as (*Z*)-α-bergamotene; Halitschke et al., 2000; Schuman et al., 2009) for which the jasmonate-mediated and herbivore-induced elicitation of emission is well characterized. (*E*)-α-Bergamotene emission from leaves has been demonstrated to attract native predators and reduce herbivore populations on plants (Halitschke et al., 2000, 2008; Kessler and Baldwin, 2001; Schuman et al., 2009, 2014, 2015).

**Figure 3:**
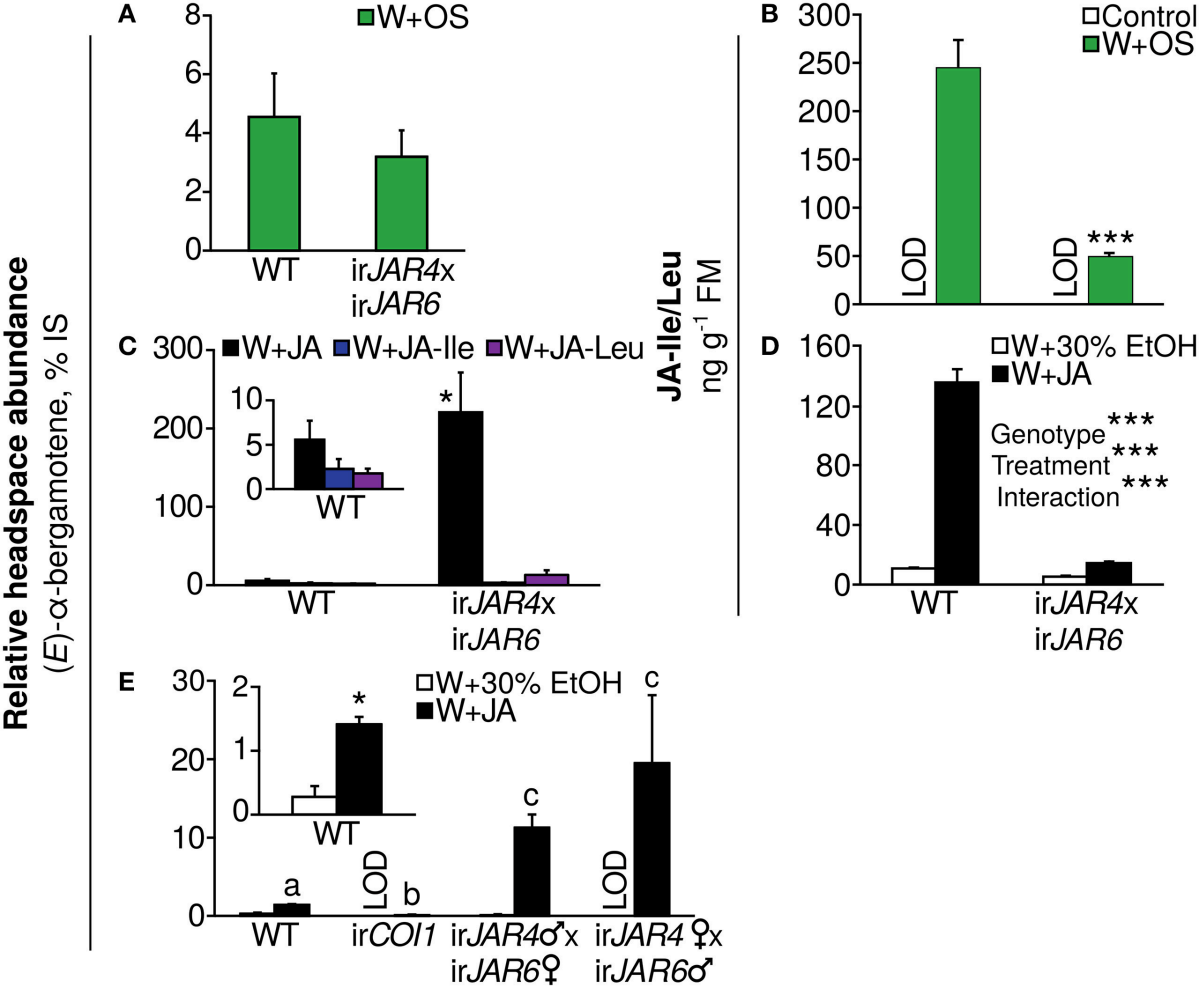
Induction of the volatile (*E*)-α-bergamotene, an effective mediator of resistance to herbivores, depends on jasmonate perception by COI1 and not on the synthesis of JA-Ile by JAR4 and JAR6. Emission of (*E*)-α-bergamotene is shown as a percentage of the internal standard peak area in the same sample (mean + SE) and was measured during peak emission 24–32 h after elicitation. **(A)** The (*E*)-α-bergamotene emission of WT and ir*JAR4*xir*JAR6* plants does not differ when leaves are subjected to simulated herbivory (W+OS, *n* = 10 plants). **(B)** The ir*JAR4*xir*JAR6* cross produces significantly less JA-Ile/JA-Leu than WT plants at peak accumulation, 1 h after plants are subjected to simulated herbivory (W+OS, *n* = 3–5 plants) (Wang et al., 2007); levels in leaves of undamaged plants were below the limit of detection (control: LOD). ^***^P < 0.001, WT vs. ir*JAR4*xir*JAR6*, Tukey's HSD *post-hoc* test following an ANOVA by genotype for a dataset including asLOX3 and irCOI1 (Table 1) after log10 transformation to achieve homogeneity of variance. **(C)** ir*JAR4*xir*JAR6* plants emit significantly more (*E*)-α-bergamotene than WT when treated with JA, but not with equimolar amounts of either JA-Ile or JA-Leu, both conjugates synthesized by JAR4 and JAR6; JA-Ile and JA-Leu are also weaker elicitors of (*E*)-α-bergamotene emission in WT plants compared to JA (*n* = 4–6 plants). ^*^Emission from ir*JAR4*xir*JAR6* differs significantly from WT plants (*P* < 0.05) in a Mann-Whitney *U*-test. **(D)** The ir*JAR4*xir*JAR6* cross produces significantly less JA-Ile/JA-Leu than WT plants at peak accumulation 3 h after plants are wounded and supplemented with JA (black bars), as well as in the wounding plus 30% ethanol solvent control (white bars); and also responds significantly less to the JA treatment (*n* = 4 plants). (Peak JA-Ile accumulation occurs later after W+JA treatment than after W+OS treatment; 1, 3, and 6 h were tested). ^***^P < 0.001 in a two-way ANOVA on data after log-transformation and mean-centering to achieve homogeneity of variance and normality; corrected for multiple testing using the false discovery rate method as part of multivariate analysis with the full measured jasmonate profile (Table 4). **(E)** Wounding of leaves and the addition of JA significantly enhances (*E*)-α-bergamotene emission from WT but not from ir*COI1* plants compared to a solvent control (30% ethanol), and this enhancement is dramatically increased in ir*JAR4*xir*JAR6* plants (*n* = 4–5 plants); results are shown separately for reciprocal crosses of the same ir*JAR4* and ir*JAR6* lines [data in **(A–C)** are from a bulk collection of these reciprocal crosses]. ^a, b^Different letters indicate significantly different emission of (*E*)-α-bergamotene (*P* < 0.05 after a Holm-Bonferroni *post-hoc* correction) in Mann-Whitney *U*-tests following a significant Kruskal-Wallis test across all genotypes; ^*^WT plants treated with W+JA emit significantly more (*E*)-α-bergamotene (*P* < 0.05 in a *t*-test followed by the Holm-Bonferroni *post-hoc* correction). LOD, below the limit of detection.

We first analyzed (*E*)-α-bergamotene emission from leaves of WT and ir*JAR4*xir*JAR6* plants after W+OS treatment and found no difference (Figure 3A) although JA-Ile accumulation was reduced by 80% in comparison to WT plants during peak accumulation, 1 h after W+OS treatment (Figure 3B, Supplementary Figure 1, Wang et al., 2007). We then tested the effect of pure jasmonates on (*E*)-α-bergamotene emission from ir*JAR4*xir*JAR6* and WT plants and found that JA was a more potent elicitor than either JA-Ile or JA-Leu, both of which conjugates are synthesized by JAR4 and JAR6; interestingly, the application of JA greatly amplified (*E*)-α-bergamotene emission from ir*JAR4*xir*JAR6* plants, to ca. 40-fold WT emission (Figure 3C) while JA-Ile/Leu accumulation in JA-treated ir*JAR4*xir*JAR6* plants was reduced by 89% compared with WT (Figure 3D, Table 4). A similar effect on (*E*)-α-bergamotene emission, 20-fold amplification, was reproduced in the reciprocal crosses of ir*JAR4* with ir*JAR6*, while emission from ir*COI1* plants remained near or below the detection limit (Figure 3E).

**Table 4:** Results of a 2-way ANOVA on hormone concentrations (mean ± SE) in leaves of the WT or ir*JAR4*xir*JAR6* genotype 3 h after wounding and treatment with 0.25 μmol JA in 20 μL 30% ethanol (W+JA), or only 30% ethanol as a control (*n* = 4 plants).

**Analyte**	**Treatment**	**Genotype**	**Mean ± SE (peak value g^−1^ FM)**	**Genotype Adj. *P***	**Treatment Adj. *P***	**Interaction Adj. *P***
JA-Ile/Leu	W+EtOH	WT	11.18 ± 0.59^a^	<**0.0001**	<**0.0001**	<**0.0001**
		ir*JAR4*xir*JAR6*	5.676 ± 0.320^a^			
	W+JA	WT	136.1 ± 8.1^a^			
		ir*JAR4*xir*JAR6*	14.77 ± 1.06^a^			
OH-JA-Ile	W+EtOH	WT	13.88 ± 3.41^a^	<**0.0001**	<**0.0001**	**0.0116**
		ir*JAR4*xir*JAR6*	4.122 ± 0.735^a^			
	W+JA	WT	205.9 ± 32.3^a^			
		ir*JAR4*xir*JAR6*	14.03 ± 2.49^a^			
JA-Met	W+EtOH	WT	0.1409 ± 0.1409^a^	**0.0015**	**0.0003**	**0.0116**
		ir*JAR4*xir*JAR6*	LOD^a^			
	W+JA	WT	13.30 ± 1.85^a^			
		ir*JAR4*xir*JAR6*	0.2123 ± 0.2123^a^			
COOH-JA-Ile	W+EtOH	WT	7.794 ± 0.604^a^	**0.0015**	0.0558	0.0778
		ir*JAR4*xir*JAR6*	5.897 ± 0.632^a^			
	W+JA	WT	14.19 ± 2.254^a^			
		ir*JAR4*xir*JAR6*	5.666 ± 0.573^a^			
JA-Phe	W+EtOH	WT	LOD^a^	**0.0030**	<**0.0001**	**0.0076**
		ir*JAR4*xir*JAR6*	LOD^a^			
	W+JA	WT	37.47 ± 1.66^a^			
		ir*JAR4*xir*JAR6*	0.7768 ± 0.5262^a^			
JA-Asn	W+EtOH	WT	0.4039 ± 0.0611^a^	**0.0151**	**0.0101**	0.8998
		ir*JAR4*xir*JAR6*	0.2162 ± 0.1905^a^			
	W+JA	WT	2.171 ± 0.381^a^			
		ir*JAR4*xir*JAR6*	0.4799 ± 0.2237^a^			
**JA-glucose**	W+EtOH	WT	LOD^a^	**0.0321**	<**0.0001**	**0.0473**
		ir*JAR4*xir*JAR6*	19.85 ± 6.80^a^			
	W+JA	WT	1010 ± 133^a^			
		ir*JAR4*xir*JAR6*	1047 ± 173^a^			
JA-Gly	W+EtOH	WT	0.2631 ± 0.1623^a^	**0.0361**	**0.0001**	0.1305
		ir*JAR4*xir*JAR6*	1.701 ± 0.480^a^			
	W+JA	WT	9.205 ± 1.01^a^			
		ir*JAR4*xir*JAR6*	12.80 ± 2.46^a^			
JA-Val	W+EtOH	WT	0.6380 ± 0.4895^a^	**0.0361**	<**0.0001**	0.4755
		ir*JAR4*xir*JAR6*	0.2151 ± 0.2151^a^			
	W+JA	WT	94.51 ± 5.64^a^			
		ir*JAR4*xir*JAR6*	7.522 ± 0.218^a^			
MeJA	W+EtOH	WT	11460 ± 514.6^b^	0.2067	<**0.0001**	0.8310
		ir*JAR4*xir*JAR6*	13440 ± 696.5^b^			
	W+JA	WT	159100 ± 10550^b^			
		ir*JAR4*xir*JAR6*	180200 ± 24440^b^			
JA-Tyr	W+EtOH	WT	LOD^a^	0.2707	**0.0001**	0.3790
		ir*JAR4*xir*JAR6*	LOD^a^			
	W+JA	WT	1.155 ± 0.386^a^			
		ir*JAR4*xir*JAR6*	0.4841 ± 0.2167^a^			
JA-Glu	W+EtOH	WT	LOD^a^	0.2707	**0.0183**	0.3790
		ir*JAR4*xir*JAR6*	LOD^a^			
	W+JA	WT	0.1886 ± 0.1077^a^			
		ir*JAR4*xir*JAR6*	0.0587 ± 0.0587^a^			
JA-Ala	W+EtOH	WT	LOD^a^	0.3556	<**0.0001**	0.4377
		ir*JAR4*xir*JAR6*	LOD^a^			
	W+JA	WT	6.836 ± 0.822^a^			
		ir*JAR4*xir*JAR6*	9.852 ± 2.513^a^			
JA-glucose formiate	W+EtOH	WT	6.487 ± 5.431^a^	0.4366	<**0.0001**	0.5237
		ir*JAR4*xir*JAR6*	19.25 ± 7.79^a^			
	W+JA	WT	21710 ± 2575^a^			
		ir*JAR4*xir*JAR6*	23370 ± 4120^a^			
JA-Gln	W+EtOH	WT	0.8359 ± 0.6437^a^	0.5901	**0.0056**	0.4377
		ir*JAR4*xir*JAR6*	1.994 ± 0.781^a^			
	W+JA	WT	10.32 ± 1.64^a^			
		ir*JAR4*xir*JAR6*	6.662 ± 1.281^a^			
12-OH-JA	W+EtOH	WT	402.8 ± 53.7^a^	0.5901	<**0.0001**	0.5782
		ir*JAR4*xir*JAR6*	401.0 ± 18.3^a^			
	W+JA	WT	8809 ± 840^a^			
		ir*JAR4*xir*JAR6*	7741 ± 1249^a^			

*Of 20 analytes, these 16 showed significant variation (false discovery rate-adjusted P-value, Adj. P < 0.05) by genotype, treatment, or the interaction of genotype and treatment: JA-glucose (in bold) was elevated while 8 other jasmonates were reduced in irJAR4xirJAR6 compared to WT. The remaining 4 analytes, salicylic acid, abscisic acid, JA-Arg, and OPDA, did not show any significant patterns, and JA-Trp and JA-His were analyzed but not detected in samples. Analytes are organized from smallest to largest adjusted P-value by genotype. The 3 h timepoint was chosen as this was the time that JA treatment-induced jasmonates were in greatest abundance (Datasheet 1). JA was not measured since it was used as a treatment. Significant adjusted P-values are in bold. JA-Ile/Leu data are also shown in Figure 3D*.

a *ng g^−1^ FM calculated using the JA-Ile internal standard*.

b *Raw peak area mg^−1^ FM, LOD: below the limit of detection*.

We asked whether other known jasmonates might be more active than JA-Ile in eliciting (*E*)-α-bergamotene emission. Interestingly, the jasmonate elicitor cis-jasmone showed the same (*E*)-α-bergamotene-inducing activity as JA in WT plants, but was less active than JA in eliciting emission from ir*JAR4*xir*JAR6* plants (ca. 20% of JA-elicited emission), and was also not active in ir*COI1* (emission not detected) (Supplementary Figure 4). Two lines which convert jasmonates to methyl jasmonate due to the ectopic expression of JASMONATE METHYL TRANSFERASE from *Arabidopsis thaliana* (s*JMT*) (Stitz et al., 2011b) had significantly lower (*E*)-α-bergamotene emission after W+OS elicitation, compared to WT plants (ca. 30% of WT levels; Supplementary Figure 5).

We also analyzed volatile emission from systemic leaves, but although these were lower and more variable, the pattern of relative emission did not vary substantially from elicited leaves (Datasheet 1).

To ensure that JA-Ile deficiency of ir*JAR4*xir*JAR6* plants was not restored by JA treatment, and to determine whether JA treatment enhanced production of another jasmonate in ir*JAR4*xir*JAR6* plants, we used LC-MS/MS to measure all jasmonates so far identified in *N. attenuata* as well as OPDA, ABA, and SA from W+JA-treated leaves (because JA was used as a treatment, it was not measured). JA-Ile and its metabolites OH-JA-Ile and COOH-JA-Ile, as well 6 other JA-AA conjugates were significantly reduced in ir*JAR4*xir*JAR6* plants compared to WT, while JA-glucose was elevated in ir*JAR4*xir*JAR6*, and levels of the other measured hormones did not differ by plant genotype (Table 4, Figure 3D, Datasheet 1).

These data indicate that an unidentified jasmonate—perhaps JA-glucose—or JA itself, is responsible for the jasmonate-mediated elicitation of (*E*)-α-bergamotene emission, and that JA-Ile may be a negative regulator of jasmonate-induced (*E*)-α-bergamotene emission.

## Discussion

Here, we used a JA-Ile-deficient cross of lines silenced in two jasmonate-isoleucine conjugating enzymes, ir*JAR4*xir*JAR6*, in comparison to lines with abrogated jasmonate biosynthesis (as*LOX3*) and perception (ir*COI1*)—all of which have been previously characterized in comparison to independently transformed lines bearing the same construct (Halitschke and Baldwin, 2003; Paschold et al., 2007; Wang et al., 2007, 2008; Stitz et al., 2011b)—to test hypotheses about the role of JA-Ile in the jasmonate-mediated defense response of the wild tobacco *N. attenuata* to herbivory. The ir*JAR4*xir*JAR6* cross produces only about 20% as much JA-Ile but the same amount of JA as WT plants after herbivore elicitation (Table 1, Wang et al., 2008); JAR4 and JAR6 transcripts were almost undetectable in Northern blots, in comparison to a strong signal for WT plants (Wang et al., 2008). We conducted reciprocal crossing between the ir*JAR4* and ir*JAR6* lines because possible maternal effects had not previously been tested for, and results from both reciprocal crosses were consistent with each other, and with the bulk collection combining both reciprocal crosses which was used for most analyses (Figure 3). All transgenic lines used in this study were in the second (T2) or third (T3) transformed generation; empty vector control plants in only the second transformed generation (T2) have been shown to be indistinguishable from WT plants in their growth and herbivore-induced transcript, hormone and metabolite production (Schwachtje et al., 2008), and thus we used WT plants as controls. Importantly, although JAR4 and JAR6 may also regulate conjugation of JA-Leu, which cannot be analytically distinguished from JA-Ile via standard mass spectrometry (MS) analysis, Wang and colleagues showed that JA-Ile application to ir*JAR4*xir*JAR6* plants was sufficient to restore gene expression (except for JAR4 and JAR6), nicotine and TPI production, and resistance to *M. sexta* larvae, to WT levels. Thus, off-target effects are unlikely.

Individual silencing of *JAR4* or *JAR6* in *N. attenuata* results in a weaker effect than the silencing of both JAR homologs, but similarly to an *S. nigrum* study, independent silencing of the *NaJAR* genes does significantly affect the accumulation of certain jasmonate-regulated secondary metabolites (Wang et al., 2007; VanDoorn et al., 2011a). Wang and colleagues showed that several defense- and growth-related genes were differentially expressed in as*LOX3* vs. ir*JAR4*xir*JAR6* lines of *N. attenuata*, and that differences in gene expression between ir*JAR4*xir*JAR6* lines and WT could be restored by JA-Ile application (except for the downregulation of the *JAR4* and *JAR6* target transcripts), indicating that JA-Ile may regulate a subset of jasmonate-mediated defense responses in *N. attenuata* (Wang et al., 2008). Together with our data, these studies indicate that JA-Ile regulates a subset of jasmonate-mediated defense in solanaceous plants. A study in *Solanum lycopersicum* using RNAi lines deficient in OPDA REDUCTASE 3 (OPR3) or JASMONATE INSENSITIVE 1 (JAI1, the homolog of COI1) showed that both the jasmonate precursor 12-oxophytodienoic acid (OPDA) and JA-Ile can mediate local defense responses, whereas JA-Ile appears to be required for systemic defense activation (Bosch et al., 2014). Thus, the specific role of JA-Ile signaling in herbivore-induced resistance may in part be explained by differences in local vs. systemic regulation of jasmonate-mediated responses. We also analyzed volatile emission from systemic leaves, but although these were lower and more variable, the pattern of relative emission was similar to elicited leaves (Datasheet 1). This likely cannot be dissected without accounting for tissue-specific expression and functions of the several JASMONATE ZIM DOMAIN (JAZ) protein repressors of jasmonate signaling, which are variable components of the SCF^COI1^-jasmonate co-receptor complex (Chini et al., 2007; Thines et al., 2007; Oh et al., 2012; Li et al., 2017), and future work along these lines may help to clarify the mechanisms of specificity in jasmonate signaling and interactions between jasmonates and other hormones.

Consistently with previous studies which quantified a few metabolites as markers of resistance, our investigation of plants' soluble metabolome revealed that JA-Ile deficiency had a relatively large effect on *N. attenuata*'s herbivore-induced soluble secondary metabolites, although less than did deficiency in jasmonate biosynthesis via *LOX3* or perception via *COI1*. In contrast, JA-Ile deficiency did not affect the volatile metabolome after herbivore elicitation. Van Poecke and Dicke (2003) also showed that *jar1-1* mutants of the brassicaceous plant *Arabidopsis thaliana*, which have reduced JA-Ile production, are able to attract parasitoids as well as WT plants, indicating that JA-Ile may not be a key jasmonate regulator of herbivore-induced plant volatile emission.

We then planted ir*JAR4*xir*JAR6* plants together with as*LOX3* and WT plants out into a field plot in the plant's native habitat in order to monitor their resistance to naturally occurring herbivores. Over more than 10 years of research at this field site and in nearby wild populations, we have found that herbivore populations on the plot reflect those in wild populations (e.g., Steppuhn et al., 2008; Kallenbach et al., 2012; Schuman et al., 2013). We found that JA-Ile deficiency had relatively small effects on susceptibility to native herbivores when compared with total jasmonate deficiency in a field experiment. The small difference in susceptibility we observed between ir*JAR4*xir*JAR6*, and WT plants, mostly resulted from a change in preference by *Trimerotropis* spp. grasshoppers: one of two generalist herbivores present in this season which we found to be affected by *N. attenuata*'s jasmonate-mediated defense. *Trimerotropis* spp. are sensitive to nicotine, which is regulated by JA-Ile (Steppuhn et al., 2004; Wang et al., 2008). It should be noted that grasshoppers are devastating herbivores in some years and so although *Trimerotropis* spp. preferences did not contribute strongly to plant damage in this year, over multiple seasons they are likely to act as a selective pressure. In contrast, another generalist, *Empoasca* sp., appear to respond to plants' jasmonate signaling capacity, and particularly to jasmonate biosynthesis, rather than to jasmonate-mediated defense (Kallenbach et al., 2012). Our data indicate that *Empoasca* sp. sense jasmonates other than JA-Ile, since this herbivore caused more damage to as*LOX3*plants, which have lower levels of all jasmonates, but not to ir*JAR4*xir*JAR6* plants, which are deficient in JA-Ile. Consistent with this inference, Kallenbach and colleagues showed that *Empoasca* sp. prefer to attack as*LOX3* and ir*COI1* vs. WT plants; while both genotypes are strongly deficient in JA, ir*COI1* is not deficient in JA-Ile biosynthesis (Table 1; Paschold et al., 2008; Kallenbach et al., 2012).

Interestingly, Vandoorn and colleagues showed that a JA-Ile-deficient ir*JAR4* line of another solanaceous plant, *Solanum nigrum*, was not more susceptible to herbivores in a study at the same field site, although a line silenced in *SnCOI1* was more susceptible (VanDoorn et al., 2011a). Vandoorn and colleagues also showed that gene regulation after herbivory changed very little in ir*JAR4 S. nigrum* plants compared to WT, and that the ir*JAR4*-regulated metabolome overlapped by about 50% with the *COI1*-regulated metabolome (VanDoorn et al., 2011a). Interestingly, *S. nigrum* ir*JAR4* plants accumulated more JA-glucose after wounding than did WT plants, but in this species the ir*JAR4* plants also accumulated more JA after wounding; these differences were eradicated after mock herbivory (W+OS) treatment which elicited larger amounts of JA and JA-glucose in both WT and ir*JAR4* (VanDoorn et al., 2011b). Vandoorn and colleagues also noted that sensitivity for JA-glucose was poor using standard LC-MS/MS analysis and instead developed a method using atmospheric pressure chemical ionization (APCI). Thus, our measurement of elevated JA-glucose in ir*JAR4*xir*JAR6 N. attenuata* plants is consistent, and intriguing, but should be interpreted cautiously.

In the field experiment, we measured herbivore-induced volatile emission from flowering plants. In the glasshouse, flowering plants have abrogated jasmonate responses, which may affect the induction of soluble secondary metabolites more than volatiles (Diezel et al., 2011; Schuman et al., 2014); and thus we used plants before flowering in glasshouse studies. However, flowering plants are more likely to experience oviposition by the herbivore/pollinator *M. sexta*, and this is the stage for which the importance of volatile-mediated defense is best understood (Kessler and Baldwin, 2001; Schuman et al., 2012; Zhou et al., 2017; Joo et al., 2018). Our volatile measurements from flowering plants in the field were consistent with the glasshouse data: while as*LOX3* plants were deficient in the emission of several leaf volatiles which were induced by herbivory in WT plants, volatile emission from ir*JAR4*xir*JAR6* plants was similar to WT. Interestingly, we found that while herbivore-induced JA and JA-Ile levels were generally lower in field-grown plants after flowering, as has been shown for plants in the glasshouse (Diezel et al., 2011), the herbivore inducibility of both jasmonates—in terms of the fold-change between basal and induced levels—was higher after flowering in field-grown plants, indicating that both may still be important regulators of induced leaf defenses after flowering.

We then used ir*JAR4*xir*JAR6* plants in further glasshouse studies employing different jasmonate elicitors, and other transgenic lines, to investigate the contribution of jasmonate signaling to herbivore-induced volatile emission more closely, focusing on the resistance compound (*E*)-α-bergamotene (initially reported as (*Z*)- α-bergamotene; Halitschke et al., 2000; Schuman et al., 2009). (*E*)-α-Bergamotene is an herbivore-induced sesquiterpene common to, but variably emitted among wild *N. attenuata* plants (Halitschke et al., 2000; Kessler and Baldwin, 2001; Schuman et al., 2009; Zhou et al., 2017), which has been shown to be regulated by jasmonate signaling (Halitschke et al., 2000, 2008; Schuman et al., 2009, 2015) and to attract native predators, resulting in the removal of up to 90% of herbivores from *N. attenuata* plants in nature (Kessler and Baldwin, 2001; Halitschke et al., 2008; Schuman et al., 2015). We found that JA-Ile deficiency as a result of *JAR4* and *JAR6* silencing had little to no effect on (*E*)-α-bergamotene emission after simulated herbivory. It has been suggested that the remaining levels (ca. 10% of WT) of JA-Ile in *A. thaliana jar1-1* mutants and *N. attenuata* ir*JAR4*xir*JAR6* plants may be sufficient for the activation of defense responses (Suza and Staswick, 2008), and other members of the *JAR* gene family could potentially be responsible for the residual 10% of JA-Ile (Staswick and Tiryaki, 2004; Wang et al., 2008). However, we show that in WT plants, JA elicited more abundant (*E*)-α-bergamotene emission than an equimolar amount of JA-Ile; and interestingly, the application of JA to ir*JAR4*xir*JAR6* plants resulted in greatly increased emission of (*E*)-α-bergamotene. Thus, JA-Ile does not appear to be a strong elicitor of (*E*)-α-bergamotene emission in *N. attenuata*. It is tempting to speculate that it may even be a negative regulator. However, Woldmariam and colleagues identified a JA-Ile hydrolase (JIH) in *N. attenuata* and surprisingly, an RNAi line deficient in JIH had greater emission of sesquiterpenes, including (*E*)-α-bergamotene. These ir*JIH* lines had ca. 5-fold the induced levels of JA-Ile as WT plants, as well as elevated JA-Ile metabolites, while other jasmonates were not affected. Together with our study, this indicates that the regulation of indirect defense responses by jasmonates cannot be fully attributed to a single compound. A study by Dinh and colleagues showed that plants deficient in a regulator of abscisic acid (ABA) metabolism, HERBIVORE-ELICITED RESPONSE 1 (HER1), had reduced levels of ABA as well as reduced accumulation of several herbivore-induced metabolites, and reduced emission of several volatiles including (*E*)-α-bergamotene (Dinh et al., 2013). ABA and JA-Ile production are correlated after simulated herbivore treatment in wild genotypes of *N. attenuata* (Schuman et al., 2009) and in *A. thaliana*, ABA has been shown to prime systemic jasmonate-mediated defense (Vos et al., 2013). This indicates that JA-Ile signaling may be involved in the separate regulation of systemic vs. local responses (Bosch et al., 2014). It should be noted that we did not identify differences in ABA accumulation between ir*JAR4*xir*JAR6* and WT plants, but the ratio of ABA:JA-Ile would differ in the two lines due to JA-Ile deficiency in ir*JAR4*xir*JAR6*. However, measurements of volatile emission from systemic leaves, though more variable and having lower signal, reflect the patterns from elicited leaves (Datasheet 1).

As an alternative to the local-systemic hypothesis, it is possible that different jasmonate signaling modules allow for the coordinated function of specific metabolites, which may not be explained only by their local vs. systemic elicitation patterns, or their volatility, and that these modules may be regulated in different ways by specific jasmonates and their interactions with other hormone signaling systems (reviewed e.g., by Robert-Seilaniantz et al., 2011). For example, RNAi-mediated silencing of JAZh de-represses the jasmonate-induced accumulation of trypsin protease inhibitors, hydroxygeranyllinalool diterpene glycosides (HGL-DTGs), and the emission of several herbivore-induced volatiles (Oh et al., 2012); all of these responses show strong local and weaker systemic induction in response to herbivory, with a distribution that meets the predictions of optimal defense theory: induced in proportion to herbivory, but also constitutively enriched in younger and reproductive tissues (Halitschke et al., 2000; van Dam et al., 2001; Heiling et al., 2010; Brütting et al., 2016; Li et al., 2017; Schäfer et al., 2017). In addition, HGL-DTGs effectively reduce the growth of *M. sexta* larvae within the background of a wild-type plant defense profile (Heiling et al., 2010), and TPIs may increase susceptibility of larvae to predation by *Geocoris* spp. predators attracted to the plant volatiles the larvae induce (Schuman et al., 2012). Interestingly, RNAi-mediated silencing of *JAZh* also suppressed nicotine accumulation (Oh et al., 2012), and the induction of nicotine is also attenuated upon plant recognition of OS from nicotine-tolerant *M. sexta* larvae (von Dahl et al., 2007). Thus, one hypothesis is that JAZh represses a functional defense module responding to attack by *M. sexta* and perhaps other nicotine-tolerant specialist herbivores (Kessler and Baldwin, 2004), and by downregulating repression by JAZh, plants might emphasize indirect over direct defense responses. It remains an open question as to what extent the regulation of JAZh and other JAZs may occur by JA-Ile-independent mechanisms.

Furthermore, it is well known that plant defense responses change with ontogeny, and optimal defense theory predicts that plants should invest more in defending first young leaves, and then reproductive tissue such as buds and flowers (Stamp, 2003; Boege and Marquis, 2005; Barton and Koricheva, 2010; Brütting et al., 2016). In *N. attenuata*, leaf jasmonate- and ethylene-mediated defense responses change drastically once plants start to flower, at least under glasshouse conditions (Diezel et al., 2011), yet jasmonate-mediated volatile emission is not abrogated in flowering plants (Schuman et al., 2014), and in fact the flowering stage is when volatile-mediated defenses, which can be highly effective against the extremely damaging specialist herbivore *M. sexta*, may be most important (Kessler and Baldwin, 2001; Kessler et al., 2010, 2015; Zhou et al., 2017). Interestingly, we show here that although JA and JA-Ile production appear to be abrogated in leaves after plants flower, their inducibility was maintained in leaves, with an even greater fold-change after induction due to lower basal levels. Because these hormone data come from field-grown plants, we cannot exclude influence of naturally occurring herbivore damage on our measurements of basal or induced levels at either growth stage. However, basal jasmonate levels in floral buds are high in comparison to leaves, and recent work on the tissue specificity of JAZ function in *N. attenuata* has provided more insight on how plants independently regulate jasmonate-mediated floral defense (Li et al., 2017). Our understanding of the complexities that determine jasmonate regulation of plant defense is likely to benefit from combining mechanistic advances with an integrative functional understanding of plant defense responses (Li et al., 2016).

## Data availability statement

All source data for this study are included in the manuscript and the supplementary files. The raw data supporting the conclusions of this manuscript will be made available by the authors, without undue reservation, to any qualified researcher.

## Author contributions

MS, SM, EG, and IB: conceptualization; MS, SM, and EG: data curation; MS, SM, EG, EM, and SG: formal analysis; MS and IB: funding acquisition; MS, SM, EG, CD, SG, and IB: investigation; MS, SM, EG, and IB: methodology; MS, SM, EG, and IB: project administration; IB: resources; MS and IB: supervision; MS, SM, EG, and EM: validation;, MS and EG: visualization; MS, SM, and EG: writing—original draft; MS, SM, EG, EM, and IB: writing—review and editing.

### Conflict of interest statement

The authors declare that the research was conducted in the absence of any commercial or financial relationships that could be construed as a potential conflict of interest.

## Supplementary material

Datasheet 1 Source data files for the article.

Supplementary Figure 1 JA and JA-Ile accumulation after mock herbivory treatment (W+OS) in WT, as*LOX3*, ir*COI1*, and ir*JAR4*xir*JAR6* plants (*n* = 3–5 plants); JA-Ile data for WT and ir*JAR4*xir*JAR6* plants is also shown in Figure 3B. ^a, b^Different letters represent statistically significant differences (*P* < 0.0001) in Tukey's HSD *post-hoc* tests following one-way ANOVAs with a false discovery rate correction for multiple analytes, after log transformation and mean-centering to achieve normality and homogeneity of variance. See also Table 1 and Figure 3B.

Supplementary Figure 2 Boxplots showing normalized peak areas of analytes which differed significantly by plant genotype (left to right: WT, red; ir*JAR4x*ir*JAR6*, green; or as*LOX3*, blue) in headspace measurements of leaves on field-grown plants before W+OS treatment (control), to accompany Table 2 (*n* = 10 plants). The lowest boxplot shows data from an unidentified green leaf volatile. ^a, b^Different letters represent statistically significant differences in Tukey *post-hoc* tests following a one-way ANOVA and corrected for multiple testing using the false discovery rate method, after log transformation and mean-centering to achieve normality and homogeneity of variance.

Supplementary Figure 3 Boxplots showing normalized peak areas of analytes which differed significantly by plant genotype (left to right: WT, red; ir*JAR4x*ir*JAR6*, green; or as*LOX3*, blue) in headspace measurements of W+OS-treated leaves on field-grown plants, to accompany Table 2 (*n* = 5–8 plants). ^a, b^Different letters represent statistically significant differences in Tukey *post-hoc* tests following a one-way ANOVA and corrected for multiple testing using the false discovery rate method, after log transformation and mean-centering to achieve normality and homogeneity of variance.

Supplementary Figure 4 A known jasmonate elicitor of volatiles, *cis*-jasmone, is not more potent than JA in eliciting (*E*)-α-bergamotene emission (*n* = 4–5 plants). Addition of either JA or an equimolar amount of *cis*-jasmone (CJ) affects (*E*)-α-bergamotene emission similarly in WT plants, but only JA and not *cis*-jasmone enhances emission in ir*JAR4*xir*JAR6* plants; results are shown separately for reciprocal crosses of the same ir*JAR4* and ir*JAR6* lines. ^a, b^Different letters indicate significantly different emission of (*E*)-α-bergamotene (*P* < 0.05 after a Holmes-Bonferroni *post-hoc* correction) in Mann-Whitney U-tests following a significant Kruskal-Wallis test across all genotypes.

Supplementary Figure 5 Methyl jasmonate is not the elicitor of (*E*)-α-bergamotene. Two independently transformed transgenic lines ectopically expressing the *Arabidopsis thaliana* jasmonate methyltransferase (s*JMT*), which converts jasmonates to methyl jasmonate, have reduced emission of (*E*)-α-bergamotene from leaves following W+OS treatment (*n* = 9–10 plants). ^*^s*JMT* lines differ significantly from WT (*P* < 0.05 after a Holmes-Bonferroni *post-hoc* correction) in Mann-Whitney *U*-tests following a significant Kruskal-Wallis test across all genotypes.

Supplementary Table 1 Deduplicated feature table from the untargeted analysis of WT, ir*JAR4*xir*JAR6*, as*LOX3*, and ir*COI1* plants in the glasshouse experiment.

Supplementary Table 2 Peak table for control leaf headspace samples from flowering plants in the field experiment.

Supplementary Table 3 Peak table for W+OS-treated leaf headspace samples from flowering plants in the field experiment.

Image 1 Contains all supplementary figures.

## References

[B1] Adam N. Erler T. Kallenbach M. Kaltenpoth M. Kunert G. Baldwin I. T. (2017). Sex ratio of mirid populations shifts in response to hostplant co-infestation or altered cytokinin signaling. J. Integr. Plant Biol. 59, 44–59. 10.1111/jipb.12507 27862998 PMC5234700

[B2] Baldwin I. T. Staszak-Kozinski L. Davidson R. (1994). Up in smoke: I. Smoke-derived germination cues for postfire annual, Nicotiana attenuata Torr. ex. Watson. J. Chem. Ecol. 2345, 2345–2371. 10.1007/BF02033207 24242811

[B3] Barton K. E. Koricheva J. (2010). The ontogeny of plant defense and herbivory: characterizing general patterns using meta-analysis. Am. Nat. 175, 481–493. 10.1086/650722 20170370

[B4] Birkett M. A. Campbell C. A. Chamberlain K. Guerrieri E. Hick A. J. Martin J. L.. (2000). New roles for cis-jasmone as an insect semiochemical and in plant defense. Proc. Natl. Acad. Sci. U.S.A. 97, 9329–9334. 10.1073/pnas.160241697 10900270 PMC16867

[B5] Boege K. Marquis R. J. (2005). Facing herbivory as you grow up: the ontogeny of resistance in plants. Trends Ecol. Evol. 20, 441–448. 10.1016/j.tree.2005.05.001 16701415

[B6] Bonaventure G. VanDoorn A. Baldwin I. T. (2011). Herbivore-associated elicitors: FAC signaling and metabolism. Trends Plant Sci. 16, 294–299. 10.1016/j.tplants.2011.01.006 21354852

[B7] Bosch M. Wright L. P. Gershenzon J. Wasternack C. Hause B. Schaller A.. (2014). Jasmonic acid and its precursor 12-oxophytodienoic acid control different aspects of constitutive and induced herbivore defenses in tomato. Plant Physiol. 166, 396–410. 10.1104/pp.114.237388 25073705 PMC4149723

[B8] Böttcher C. Pollmann S. (2009). Plant oxylipins: plant responses to 12-oxo-phytodienoic acid are governed by its specific structural and functional properties. FEBS J. 276, 4693–4704. 10.1111/j.1742-4658.2009.07195.x 19663904

[B9] Bruce T. J. Matthes M. C. Chamberlain K. Woodcock C. M. Mohib A. Webster B.. (2008). cis-Jasmone induces Arabidopsis genes that affect the chemical ecology of multitrophic interactions with aphids and their parasitoids. Proc. Natl. Acad. Sci. U.S.A. 105, 4553–4558. 10.1073/pnas.0710305105 18356298 PMC2290791

[B10] Brütting C. Schäfer M. Vanková R. Gase K. Baldwin I. T. Meldau S. (2016). Changes in cytokinins are sufficient to alter developmental patterns of defense metabolites in *Nicotiana attenuata*. Plant J. 89, 15–30. 10.1111/tpj.13316 27557345 PMC5245775

[B11] Chini A. Fonseca S. Fernández G. Adie B. Chico J. M. Lorenzo O.. (2007). The JAZ family of repressors is the missing link in jasmonate signalling. Nature 448, 666–671. 10.1038/nature06006 17637675

[B12] Dicke M. Baldwin I. T. (2010). The evolutionary context for herbivore-induced plant volatiles: beyond the “cry for help.” Trends Plant Sci. 15, 167–175. 10.1016/j.tplants.2009.12.002 20047849

[B13] Diezel C. Allmann S. Baldwin I. T. (2011). Mechanisms of optimal defense patterns in *Nicotiana attenuata*: flowering attenuates herbivory-elicited ethylene and jasmonate signaling(F). J. Integr. Plant Biol. 53, 971–983. 10.1111/j.1744-7909.2011.01086.x 22054509

[B14] Dinh S. T. Baldwin I. T. Galis I. (2013). The HERBIVORE ELICITOR-REGULATED1 (HER1) gene enhances abscisic acid levels and defenses against herbivores in *Nicotiana attenuata* plants. Plant Physiol. 162, 2106–2124. 10.1104/pp.113.221150 23784463 PMC3729786

[B15] Erb M. Glauser G. (2010). Family business: multiple members of major phytohormone classes orchestrate plant stress responses. Chemistry 16, 10280–10289. 10.1002/chem.201001219 20648494

[B16] Erb M. Meldau S. Howe G. A. (2012). Role of phytohormones in insect-specific plant reactions. Trends Plant Sci. 17, 250–259. 10.1016/j.tplants.2012.01.003 22305233 PMC3346861

[B17] Fonseca S. Chini A. Hamberg M. Adie B. Porzel A. Kramell R.. (2009). (+)-7-iso-Jasmonoyl-L-isoleucine is the endogenous bioactive jasmonate. Nat. Chem. Biol. 5, 344–350. 10.1038/nchembio.161 19349968

[B18] Gaquerel E. Heiling S. Schoettner M. Zurek G. Baldwin I. T. (2010). Development and validation of a liquid chromatography-electrospray ionization-time-of-flight mass spectrometry method for induced changes in *Nicotiana attenuata* leaves during simulated herbivory. J. Agric. Food Chem. 58, 9418–9427. 10.1021/jf1017737 20701244

[B19] Gaquerel E. Steppuhn A. Baldwin I. T. (2012). *Nicotiana attenuata* a-DOX1 through its production of 2-HOT is required for intact plant defense expression against attack from *Manduca sexta* larvae. New Phytol. 196, 574–585. 10.1111/j.1469-8137.2012.04286.x 22937952

[B20] Gaquerel E. Weinhold A. Baldwin I. T. (2009). Molecular interactions between the specialist herbivore *Manduca sexta* (Lepidoptera, Sphigidae) and its natural host *Nicotiana attenuata*. VIII. An unbiased GCxGC-ToFMS analysis of the plant's elicited volatile emissions. Plant Physiol. 149, 1408–1423. 10.1104/pp.108.130799 19136568 PMC2649405

[B21] De Geyter N. Gholami A. Goormachtig S. Goossens A. (2012). Transcriptional machineries in jasmonate-elicited plant secondary metabolism. Trends Plant Sci. 17, 349–359. 10.1016/j.tplants.2012.03.001 22459758

[B22] Halitschke R. Baldwin I. T. (2003). Antisense LOX expression increases herbivore performance by decreasing defense responses and inhibiting growth-related transcriptional reorganization in *Nicotiana attenuata*. Plant J. 36, 794–807. 10.1046/j.1365-313X.2003.01921.x 14675445

[B23] Halitschke R. Gase K. Hui D. Schmidt D. D. Baldwin I. T. (2003). Molecular interactions between the specialist herbivore *Manduca sexta* (Lepidoptera, Sphingidae) and its natural host *Nicotiana attenuata*. VI. Microarray analysis reveals that most herbivore-specific transcriptional changes are mediated by fatty acid-amino acid conjugates. Plant Physiol. 131, 1894–1902. 10.1104/pp.102.018184 12692348 PMC166945

[B24] Halitschke R. Keßler A. Kahl J. Lorenz A. Baldwin I. T. (2000). Ecophysiological comparison of direct and indirect defenses in *Nicotiana attenuata*. Oecologia 124, 408–417. 10.1007/s004420000389 28308780

[B25] Halitschke R. Schittko U. Pohnert G. Boland W. Baldwin I. T. (2001). Molecular interactions between the specialist herbivore *Manduca sexta* (Lepidoptera, Sphingidae) and its natural host *Nicotiana attenuata*. III. Fatty acid-amino acid conjugates in herbivore oral secretions are necessary and sufficient for herbivore-specific responses. Plant Physiol. 125, 711–717. 10.1104/pp.125.2.711 11161028 PMC64872

[B26] Halitschke R. Stenberg J. A. Kessler D. Kessler A. Baldwin I. T. (2008). Shared signals - “alarm calls” from plants increase apparency to herbivores and their enemies in nature. Ecol. Lett. 11, 24–34. 10.1111/j.1461-0248.2007.01123.x 17961175

[B27] Heiling S. Schuman M. C. Schoettner M. Mukerjee P. Berger B. Schneider B.. (2010). Jasmonate and ppHsystemin regulate key Malonylation steps in the biosynthesis of 17-hydroxygeranyllinalool diterpene glycosides, an abundant and effective direct defense against herbivores in *Nicotiana attenuata*. Plant Cell 22, 273–292. 10.1105/tpc.109.071449 20081114 PMC2828710

[B28] Helder H. Miersch O. Vreugdenhil D. Sembdner G. (1993). Occurrence of hydroxylated jasmonic acids in leaflets of Solanum demissum plants grown under long- and short-day conditions. Physiol. Plant. 88, 647–653. 10.1111/j.1399-3054.1993.tb01384.x 28741774

[B29] Joo Y. Schuman M. C. Goldberg J. K. Kim S.-G. Yon F. Brütting C. (2018). Herbivore-induced volatile blends with both “fast” and “slow” components provide robust indirect defence in nature. Funct. Ecol. 32, 136–149. 10.1111/1365-2435.12947

[B30] Kallenbach M. Alagna F. Baldwin I. T. Bonaventure G. (2010). *Nicotiana attenuata* SIPK, WIPK, NPR1, and fatty acid-amino acid conjugates participate in the induction of jasmonic acid biosynthesis by affecting early enzymatic steps in the pathway. Plant Physiol. 152, 96–106. 10.1104/pp.109.149013 19897603 PMC2799349

[B31] Kallenbach M. Bonaventure G. Gilardoni P. A. Wissgott A. Baldwin I. T. (2012). Empoasca leafhoppers attack wild tobacco plants in a jasmonate-dependent manner and identify jasmonate mutants in natural populations. Proc. Natl. Acad. Sci. U.S.A. 109, E1548–E1557. 10.1073/pnas.1200363109 22615404 PMC3386116

[B32] Kallenbach M. Oh Y. Eilers E. J. Veit D. Baldwin I. T. Schuman M. C. (2014). A robust, simple, high-throughput technique for time- resolved plant volatile analysis in field experiments. Plant J. 78, 1060–1072. 10.1111/tpj.12523 24684685 PMC4190661

[B33] Kallenbach M. Veit D. Eilers E. J. Schuman M. C. (2015). Application of silicone tubing for robust, simple, high-throughput, and time-resolved analysis of plant volatiles in field experiments. Bio Protocol 5, 1–8. 10.21769/BioProtoc.1391 29085860 PMC5660617

[B34] Katsir L. Schilmiller A. L. Staswick P. E. He S. Y. Howe G. A. (2008). COI1 is a critical component of a receptor for jasmonate and the bacterial virulence factor coronatine. Proc. Natl. Acad. Sci. U.S.A. 105, 7100–7105. 10.1073/pnas.0802332105 18458331 PMC2383947

[B35] Kessler A. Baldwin I. T. (2001). Defensive function of herbivore-induced plant volatile emissions in nature. Science 291, 2141–2144. 10.1126/science.291.5511.2141 11251117

[B36] Kessler A. Baldwin I. T. (2004). Herbivore-induced plant vaccination. Part, I. The orchestration of plant defenses in nature and their fitness consequences in the wild tobacco *Nicotiana attenuata*. Plant J. 38, 639–649. 10.1111/j.1365-313X.2004.02076.x 15125770

[B37] Kessler A. Halitschke R. Baldwin I. T. (2004). Silencing the jasmonate cascade: induced plant defenses and insect populations. Science 305, 665–668. 10.1126/science.1096931 15232071

[B38] Kessler D. Diezel C. Baldwin I. T. (2010). Changing pollinators as a means of escaping herbivores. Curr. Biol. 20, 237–242. 10.1016/j.cub.2009.11.071 20096581

[B39] Kessler D. Kallenbach M. Diezel C. Rothe E. Murdock M. Baldwin I. T. (2015). How scent and nectar influence floral antagonists and mutualists. Elife:7641. 10.7554/eLife.07641 26132861 PMC4530224

[B40] Krügel T. Lim M. Gase K. Halitschke R. Baldwin I. T. (2002). Agrobacterium-mediated transformation of *Nicotiana attenuata*, a model ecological expression system. Chemoecology 12, 177–183. 10.1007/PL00012666

[B41] Kuhl C. Tautenhahn R. Böttcher C. Larson T. R. Neumann S. (2012). CAMERA: an integrated strategy for compound spectra extraction and annotation of liquid chromatography/mass spectrometry data sets. Anal. Chem. 84:283–289. 10.1021/ac202450g 22111785 PMC3658281

[B42] Li D. Baldwin I. T. Gaquerel E. (2016). Beyond the canon: within-plant and population-level heterogeneity in jasmonate signaling engaged by plant-insect interactions. Plants 5:14. 10.3390/plants5010014 27135234 PMC4844416

[B43] Li R. Wang M. Wang Y. Schuman M. C. Weinhold A. Schäfer M.. (2017). Flower-specific jasmonate signaling regulates constitutive floral defenses in wild tobacco. Proc. Natl. Acad. Sci. U.S.A. 114, E7205–E7214. 10.1073/pnas.1703463114 28784761 PMC5576791

[B44] McGale E. Diezel C. Schuman M. C. Baldwin I. T. (2018). Cry1Ac production is costly for native plants attacked by non-Cry1Ac-targeted herbivores in the field. New Phytol. 10.1111/nph.15207 29754424

[B45] Meldau S. Wu J. Baldwin I. T. (2009). Silencing two herbivory-activated MAP kinases, SIPK and WIPK, does not increase *Nicotiana attenuata'*s susceptibility to herbivores in the glasshouse and in nature. New Phytol. 181, 161–173. 10.1111/j.1469-8137.2008.02645.x 19076722

[B46] Miersch O. Neumerkel J. Dippe M. Stenzel I. Wasternack C. (2008). Hydroxylated jasmonates are commonly occurring metabolites of jasmonic acid and contribute to a partial switch-off in jasmonate signaling. New Phytol. 177, 114–127. 10.1111/j.1469-8137.2007.02252.x 17995915

[B47] Mithöfer A. Boland W. (2012). Plant defense against herbivores: chemical aspects. Annu. Rev. Plant Biol. 63, 431–450. 10.1146/annurev-arplant-042110-103854 22404468

[B48] Nakamura Y. Mithoefer A. Kombrink E. Boland W. Hamamoto S. Uozumi N. (2011). 12-hydroxyjasmonic acid glucoside is a COI1-JAZs independent activator of leaf closing movement in *Samanea saman*. Plant Physiol. 155, 1226–1236. 10.1104/pp.110.168617 21228101 PMC3046581

[B49] Oh Y. Baldwin I. T. Gális I. Galis I. (2012). NaJAZh regulates a subset of defense responses against herbivores and spontaneous leaf necrosis in *Nicotiana attenuata* plants. Plant Physiol. 159, 769–788. 10.1104/pp.112.193771 22496510 PMC3375940

[B50] Paschold A. Bonaventure G. Kant M. R. Baldwin I. T. (2008). Jasmonate perception regulates jasmonate biosynthesis and JA-Ile metabolism: the case of COI1 in *Nicotiana attenuata*. Plant Cell Physiol. 49, 1165–1175. 10.1093/pcp/pcn091 18559356

[B51] Paschold A. Halitschke R. Baldwin I. T. (2007). Co (i) -ordinating defenses: NaCOI1 mediates herbivore- induced resistance in *Nicotiana attenuata* and reveals the role of herbivore movement in avoiding defenses. Plant J. 51, 79–91. 10.1111/j.1365-313X.2007.03119.x 17561925

[B52] Radhika V. Kost C. Mithöfer A. Boland W. (2010). Regulation of extrafloral nectar secretion by jasmonates in lima bean is light dependent. Proc. Natl. Acad. Sci. U.S.A. 107, 17228–17233. 10.1073/pnas.1009007107 20855624 PMC2951398

[B53] Ribot C. Zimmerli C. Farmer E. E. Reymond P. Poirier Y. (2008). Induction of the Arabidopsis PHO1; H10 gene by 12-oxo-phytodienoic acid but not jasmonic acid via a CORONATINE INSENSITIVE1-dependent pathway. Plant Physiol. 147, 696–706. 10.1104/pp.108.119321 18434606 PMC2409032

[B54] Robert-Seilaniantz A. Grant M. Jones J. D. (2011). Hormone crosstalk in plant disease and defense: more than just jasmonate-salicylate antagonism. Annu. Rev. Phytopathol. 49, 317–343. 10.1146/annurev-phyto-073009-114447 21663438

[B55] Schäfer M. Brütting C. Xu S. Ling Z. Steppuhn A. Baldwin I. T.. (2017). NaMYB8 regulates distinct, optimally distributed herbivore defense traits. J. Integr. Plant Biol. 59, 844–850. 10.1111/jipb.12593 28843024

[B56] Schäfer M. Fischer C. Meldau S. Seebald E. Oelmüller R. Baldwin I. T. (2011). Lipase activity in insect oral secretions mediates defense responses in Arabidopsis. Plant Physiol. 156, 1520–1534. 10.1104/pp.111.173567 21546453 PMC3135923

[B57] Schittko U. Hermsmeier D. Baldwin I. T. (2001). Molecular interactions between the specialist herbivore *Manduca sexta* (Lepidoptera, Sphingidae) and its natural host *Nicotiana attenuata*. II. Accumulation of plant mRNAs in response to insect-derived cues. Plant Physiol. 125, 701–710. 10.1104/pp.125.2.701 11161027 PMC64871

[B58] Schittko U. Preston C. A. Baldwin I. T. (2000). Eating the evidence? *Manduca sexta* larvae can not disrupt specific jasmonate induction in *Nicotiana attenuata* by rapid consumption. Planta 210, 343–346. 10.1007/PL00008143 10664142

[B59] Schuman M. C. Allmann S. Baldwin I. T. (2015). Plant defense phenotypes determine the consequences of volatile emission for individuals and neighbors. Elife 4:e04490. 10.7554/eLife.04490 25873033 PMC4397498

[B60] Schuman M. C. Baldwin I. T. (2016). The layers of plant responses to insect herbivores. Annu. Rev. Entomol. 61, 373–394. 10.1146/annurev-ento-010715-023851 26651543

[B61] Schuman M. C. Barthel K. Baldwin I. T. (2012). Herbivory-induced volatiles function as defenses increasing fitness of the native plant *Nicotiana attenuata* in nature. Elife 2012:e00007 10.7554/eLife.00007 PMC3466783 23066503

[B62] Schuman M. C. Heinzel N. Gaquerel E. Svatos A. Baldwin I. T. (2009). Polymorphism in jasmonate signaling partially accounts for the variety of volatiles produced by *Nicotiana attenuata* plants in a native population. New Phytol. 183, 1134–1148. 10.1111/j.1469-8137.2009.02894.x 19538549

[B63] Schuman M. C. Kessler D. Baldwin I. T. (2013). Ecological observations of native Geocoris pallens and G. punctipes populations in the great basin desert of Southwestern Utah. Psyche 2013:465108 10.1155/2013/465108 PMC4185340 25298571

[B64] Schuman M. C. Palmer-Young E. P. C. Schmidt A. Gershenzon J. Baldwin I. T. (2014). Ectopic TPS expression enhances sesquiterpene emission in *Nicotiana attenuata* without altering defense or development of transgenic plants or neighbors. Plant Physiol. 166, 779–797. 10.1104/pp.114.247130 25187528 PMC4190577

[B65] Schwachtje J. Kutschbach S. Baldwin I. T. (2008). Reverse genetics in ecological research. PLoS ONE 3:e1543. 10.1371/journal.pone.0001543 18253491 PMC2212111

[B66] Seo H. S. Song J. T. Cheong J. Lee Y. Lee Y. Hwang I.. (2001). Jasmonic acid carboxyl methyltransferase : a key enzyme for jasmonate-regulated plant responses. Proc. Natl. Acad. Sci. U.S.A. 98, 4788–4793. 10.1073/pnas.081557298 11287667 PMC31912

[B67] Sheard L. B. Tan X. Mao H. Withers J. Ben-nissan G. Hinds T. R.. (2010). Jasmonate perception by inositol-phosphate-potentiated COI1–JAZ co-receptor. Nature 468, 400–407. 10.1038/nature09430 20927106 PMC2988090

[B68] Smith C. A Want E. J. O'Maille G. Abagyan R. Siuzdak G. (2006). XCMS: processing mass spectrometry data for metabolite profiling using nonlinear peak alignment, matching, and identification. Anal. Chem. 78, 779–787. 10.1021/ac051437y 16448051

[B69] Snoeren T. A. Van Poecke R. M. Dicke M. (2009). Multidisciplinary approach to unravelling the relative contribution of different oxylipins in indirect defense of *Arabidopsis thaliana*. J. Chem. Ecol. 35, 1021–1031. 10.1007/s10886-009-9696-3 19798534 PMC2759439

[B70] Stamp N. (2003). Out of the quagmire of plant defense hypotheses. Q. Rev. Biol. 78, 23–55. 10.1086/367580 12661508

[B71] Staswick P. E. Tiryaki I. (2004). The oxylipin signal jasmonic acid is activated by an enzyme that conjugates it to isoleucine in Arabidopsis. Plant Cell 16, 2117–2127. 10.1105/tpc.104.023549 15258265 PMC519202

[B72] Staswick P. E. Yuen G. Y. Lehman C. C. (1998). Jasmonate signaling mutants of Arabidopsis are susceptible to the soil fungus *Pythium irregulare*. Plant J. 15, 747–754. 10.1046/j.1365-313X.1998.00265.x 9807813

[B73] Steppuhn A. Gase K. Krock B. Halitschke R. Baldwin I. T. (2004). Nicotine's defensive function in nature. PLoS Biol. 2:e217. 10.1371/journal.pbio.0020217 15314646 PMC509292

[B74] Steppuhn A. Schuman M. C. Baldwin I. T. (2008). Silencing jasmonate signalling and jasmonate-mediated defences reveals different survival strategies between two *Nicotiana attenuata* accessions. Mol. Ecol. 17, 3717–3732. 10.1111/j.1365-294X.2008.03862.x 18662222

[B75] Stintzi A. Weber H. Reymond P. Browse J. Farmer E. E. (2001). Plant defense in the absence of jasmonic acid : the role of cyclopentenones. Proc. Natl. Acad. Sci. U.S.A. 98, 12837–12842. 10.1073/pnas.211311098 11592974 PMC60140

[B76] Stitz M. Baldwin I. T. Gaquerel E. (2011a). Diverting the flux of the JA pathway in *Nicotiana attenuata* compromises the plant's defense metabolism and fitness in nature and glasshouse. PLoS ONE 6:e25925. 10.1371/journal.pone.0025925 22022469 PMC3189938

[B77] Stitz M. Gase K. Baldwin I. T. Gaquerel E. (2011b). Ectopic expression of AtJMT in *Nicotiana attenuata*: creating a metabolic sink has tissue-specific consequences for the jasmonate metabolic network and silences downstream gene expression. Plant Physiol. 157, 341–354. 10.1104/pp.111.178582 21753114 PMC3165883

[B78] Suza W. P. Staswick P. E. (2008). The role of JAR1 in jasmonoyl-l-isoleucine production during Arabidopsis wound response. Planta 227, 1221–1232. 10.1007/s00425-008-0694-4 18247047

[B79] Swiatek A. Van Dongen W. Esmans E. L. Van Onckelen H. (2004). Metabolic fate of jasmonates in tobacco bright yellow-2 cells. Plant Physiol. 135, 161–172. 10.1104/pp.104.040501 15133155 PMC429344

[B80] Taki N. (2005). 12-Oxo-phytodienoic acid triggers expression of a distinct set of genes and plays a role in wound-induced gene expression in Arabidopsis. Plant Physiol. 139, 1268–1283. 10.1104/pp.105.067058 16258017 PMC1283764

[B81] Tautenhahn R. Böttcher C. Neumann S. (2008). Highly sensitive feature detection for high resolution LC/MS. BMC Bioinformatics 9:504. 10.1186/1471-2105-9-504 19040729 PMC2639432

[B82] Thines B. Katsir L. Melotto M. Niu Y. Mandaokar A. Liu G.. (2007). JAZ repressor proteins are targets of the SCF complex during jasmonate signalling. Nature 448, 661–665. 10.1038/nature05960 17637677

[B83] Tholl D. Boland W. Hansel A. Loreto F. Röse U. S. Schnitzler J. P. (2006). Practical approaches to plant volatile analysis. Plant J. 45, 540–560. 10.1111/j.1365-313X.2005.02612.x 16441348

[B84] van Dam N. M. Horn M. Mares M. Baldwin I. T. (2001). Ontogeny constrains systemic protease inhibitor response in *Nicotiana attenuata*. J. Chem. Ecol. 27, 547–568. 10.1023/A:1010341022761 11441445

[B85] VanDoorn A. Bonaventure G. Rogachev I. Aharoni A. Baldwin I. T. (2011a). JA-Ile signaling in *Solanum nigrum* is not required for defense responses in nature. Plant Cell Environ. 34, 2159–2171. 10.1111/j.1365-3040.2011.02412.x 21883286

[B86] VanDoorn A. Bonaventure G. Schmidt D. D. Baldwin I. T. (2011b). Regulation of jasmonate metabolism and activation of systemic signaling in *Solanum nigrum*: COI1 and JAR4 play overlapping yet distinct roles. New Phytol. 190, 640–652. 10.1111/j.1469-8137.2010.03622.x 21284648

[B87] Van Poecke R. Dicke M. (2003). Signal transduction downstream of salicylic and jasmonic acid in herbivory-induced parasitoid attraction by Arabidopsis is independent of JAR1 and NPR1. Plant. Cell Environ. 26, 1541–1548. 10.1046/j.1365-3040.2003.01078.x

[B88] von Dahl C. C. Winz R. A. Halitschke R. Kühnemann F. Gase K. Baldwin I. T. (2007). Tuning the herbivore-induced ethylene burst: the role of transcript accumulation and ethylene perception in *Nicotiana attenuata*. Plant J. 51, 293–307. 10.1111/j.1365-313X.2007.03142.x 17559506

[B89] Vos I. A. Verhage A. Schuurink R. C. Watt L. G. Pieterse C. M. Van Wees S. C. (2013). Onset of herbivore-induced resistance in systemic tissue primed for jasmonate-dependent defenses is activated by abscisic acid. Front. Plant Sci. 4:539. 10.3389/fpls.2013.00539 24416038 PMC3874679

[B90] Walter A. Mazars C. Maitrejean M. Hopke J. Ranjeva R. Boland W.. (2007). Structural requirements of jasmonates and synthetic analogues as inducers of Ca^2+^ signals in the nucleus and the cytosol of plant cells. Angew. Chemie Int. Ed. Engl. 46, 4783–4785. 10.1002/anie.200604989 17487903

[B91] Wang L. Allmann S. Wu J. Baldwin I. T. (2008). Comparisons of LIPOXYGENASE3- and JASMONATE-RESISTANT4/6-silenced plants reveal that jasmonic acid and jasmonic acid-amino acid conjugates play different roles in herbivore resistance. Plant Physiol. 146, 904–915. 10.1104/pp.107.109264 18065562 PMC2259097

[B92] Wang L. Halitschke R. Berg A. Harnisch F. Baldwin I. T. (2007). Independently silencing two JAR family members impairs levels of trypsin proteinase inhibitors but not nicotine. Planta 226, 159–167. 10.1007/s00425-007-0477-3 17273867

[B93] Wasternack C. (2007). Jasmonates: an update on biosynthesis, signal transduction and action in plant stress response, growth and development. Ann. Bot. 100, 681–697. 10.1093/aob/mcm079 17513307 PMC2749622

[B94] Wasternack C. Hause B. (2013). Jasmonates: biosynthesis, perception, signal transduction and action in plant stress response, growth and development. An update to the 2007 review in Annals of Botany. Ann. Bot. 111, 1021–1058. 10.1093/aob/mct067 23558912 PMC3662512

[B95] Wasternack C. Strnad M. (2016). Jasmonate signaling in plant stress responses and development–active and inactive compounds. N. Biotechnol. 25, 604–613. 10.1016/j.nbt.2015.11.001 26581489

[B96] Xia J. Psychogios N. Young N. Wishart D. S. (2009). MetaboAnalyst: a web server for metabolomic data analysis and interpretation. Nucleic Acids Res. 37, W652–W660. 10.1093/nar/gkp356 19429898 PMC2703878

[B97] Xia J. Sinelnikov I. V. Han B. Wishart D. S. (2015). MetaboAnalyst 3.0–making metabolomics more meaningful. Nucleic Acids Res. 43(W1):W251–W257. 10.1093/nar/gkv380 25897128 PMC4489235

[B98] Xia J. Wishart D. S. (2016). Using metaboanalyst 3.0 for comprehensive metabolomics data analysis. Curr. Protoc. Bioinform. 55, 14.10.1–14.10.91. 10.1002/cpbi.11 27603023

[B99] Xu L. Liu F. Lechner E. Genschik P. Crosby W. L. Peng W.. (2002). The SCF COI1 ubiquitin-ligase complexes are required for jasmonate response in Arabidopsis. Plant Cell 14, 1919–1935. 10.1105/tpc.003368 12172031 PMC151474

[B100] Yoshihara T. Omer E. S. A. Koshino H. Sakamura S. Kikuta Y. Koda Y. (1989). Structure of a tuber-inducing stimulus from potato leaves (*Solanum Tuberosum*, L.). Agric. Biol. Chem. 53, 2835–2837. 10.1271/bbb1961.53.2835

[B101] Zhou W. Kügler A. McGale E. Haverkamp A. Knaden M. Guo H.. (2017). Tissue-specific emission of (E)-α-bergamotene helps resolve the dilemma when pollinators are also herbivores. Curr. Biol. 27, 1336–1341. 10.1016/j.cub.2017.03.017 28434859

