# Supplementary figures and images for: The Active Jasmonate JA-Ile Regulates a Specific Subset of Plant Jasmonate-Mediated Resistance to Herbivores in Nature
Source: Front Plant Sci. 2018 Jun 14;9:787. doi: 10.3389/fpls.2018.00787 (PMC6010948; doi:10.3389/fpls.2018.00787)

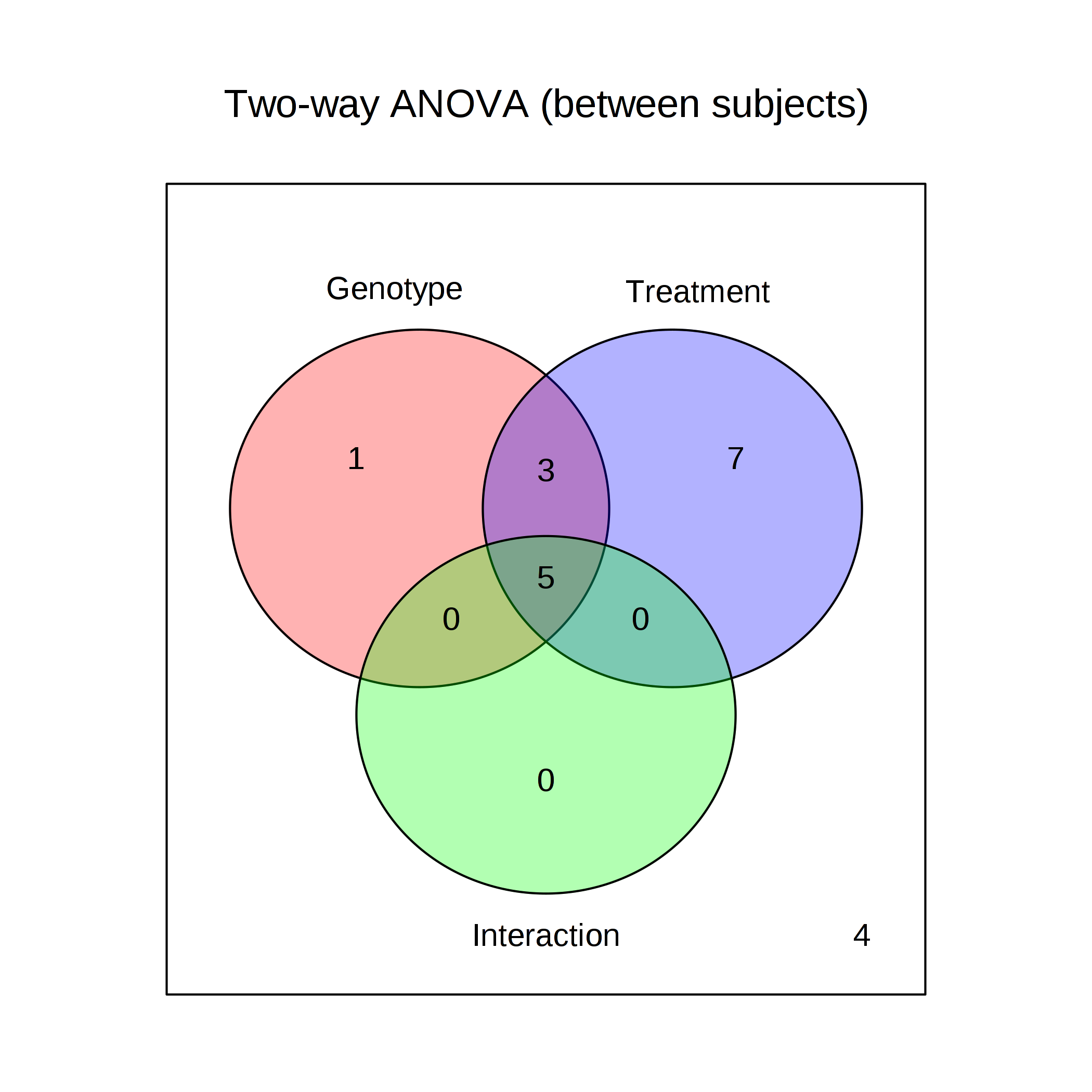

Supplement: Datasheet 1 — Source data files for the article. [file Data_Sheet_1.zip › Schuman2018_FPS_Source_Files/Metaboanalyst_W+EtOH_W+JA/aov2_0_dpi300.png]

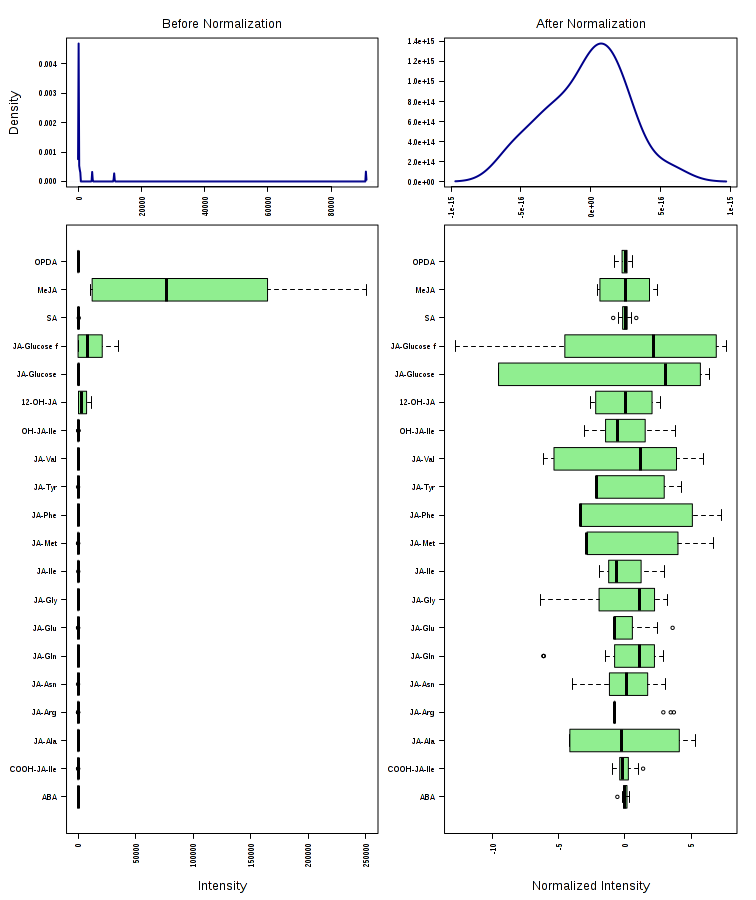

Supplement: Datasheet 1 — Source data files for the article. [file Data_Sheet_1.zip › Schuman2018_FPS_Source_Files/Metaboanalyst_W+EtOH_W+JA/norm_0_dpi72.png]

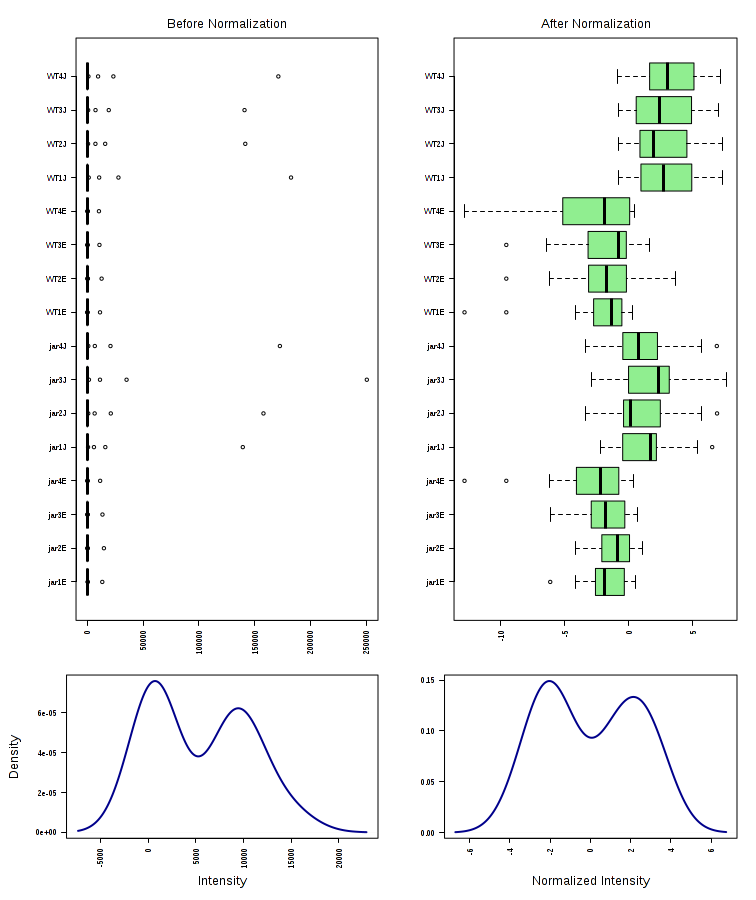

Supplement: Datasheet 1 — Source data files for the article. [file Data_Sheet_1.zip › Schuman2018_FPS_Source_Files/Metaboanalyst_W+EtOH_W+JA/snorm_0_dpi72.png]

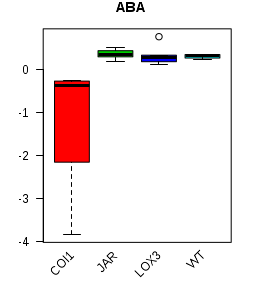

Supplement: Datasheet 1 — Source data files for the article. [file Data_Sheet_1.zip › Schuman2018_FPS_Source_Files/Metaboanalyst_W+OS_phytohormones/ABA_dpi72.png]

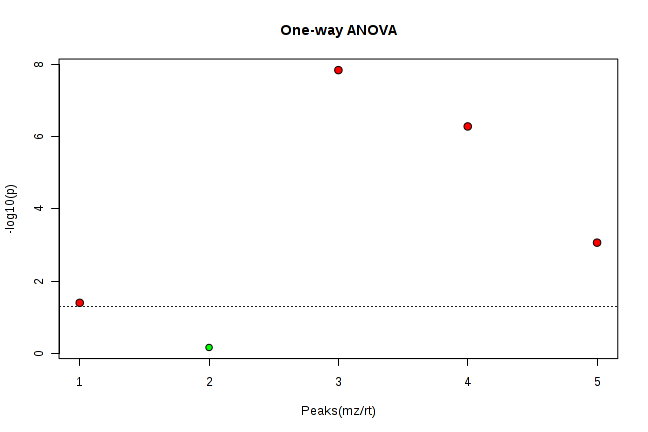

Supplement: Datasheet 1 — Source data files for the article. [file Data_Sheet_1.zip › Schuman2018_FPS_Source_Files/Metaboanalyst_W+OS_phytohormones/aov_0_dpi72.png]

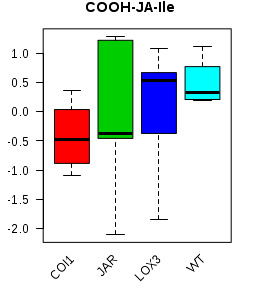

Supplement: Datasheet 1 — Source data files for the article. [file Data_Sheet_1.zip › Schuman2018_FPS_Source_Files/Metaboanalyst_W+OS_phytohormones/COOH-JA-Ile_dpi72.png]

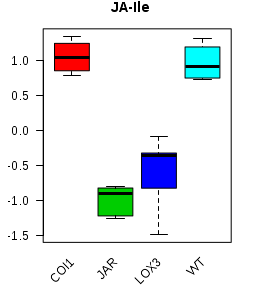

Supplement: Datasheet 1 — Source data files for the article. [file Data_Sheet_1.zip › Schuman2018_FPS_Source_Files/Metaboanalyst_W+OS_phytohormones/JA-Ile_dpi72.png]

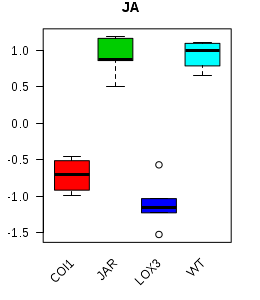

Supplement: Datasheet 1 — Source data files for the article. [file Data_Sheet_1.zip › Schuman2018_FPS_Source_Files/Metaboanalyst_W+OS_phytohormones/JA_dpi72.png]

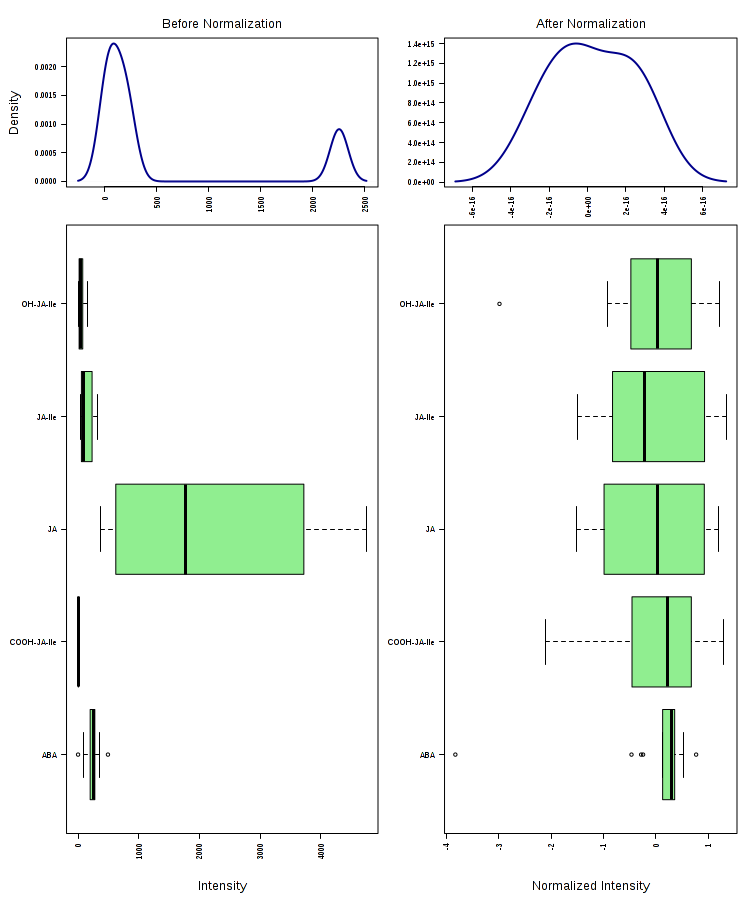

Supplement: Datasheet 1 — Source data files for the article. [file Data_Sheet_1.zip › Schuman2018_FPS_Source_Files/Metaboanalyst_W+OS_phytohormones/norm_0_dpi72.png]

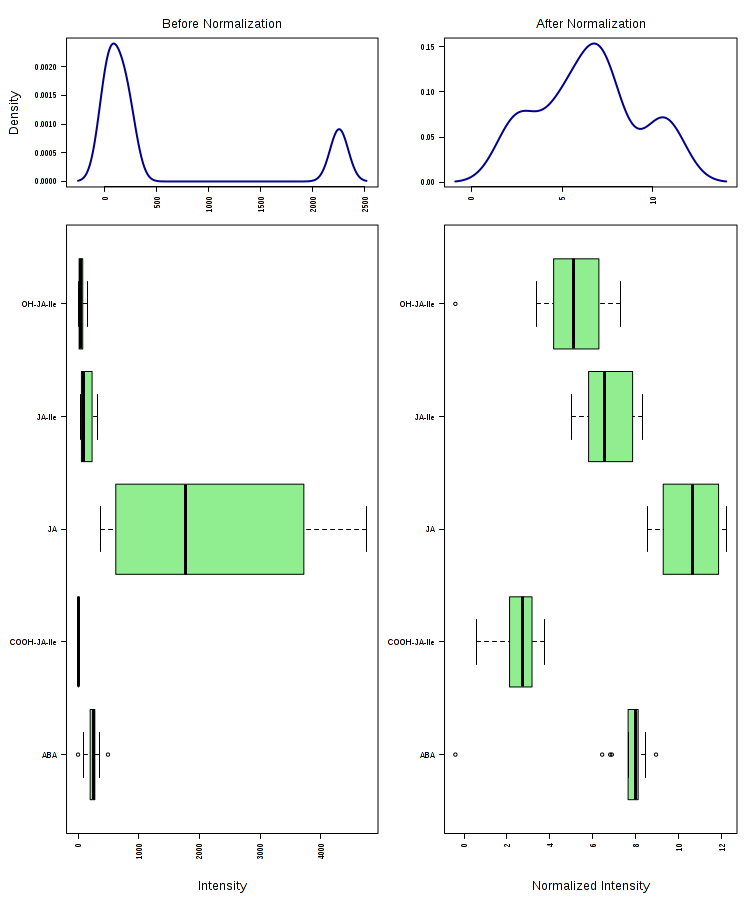

Supplement: Datasheet 1 — Source data files for the article. [file Data_Sheet_1.zip › Schuman2018_FPS_Source_Files/Metaboanalyst_W+OS_phytohormones/norm_1_dpi72.png]

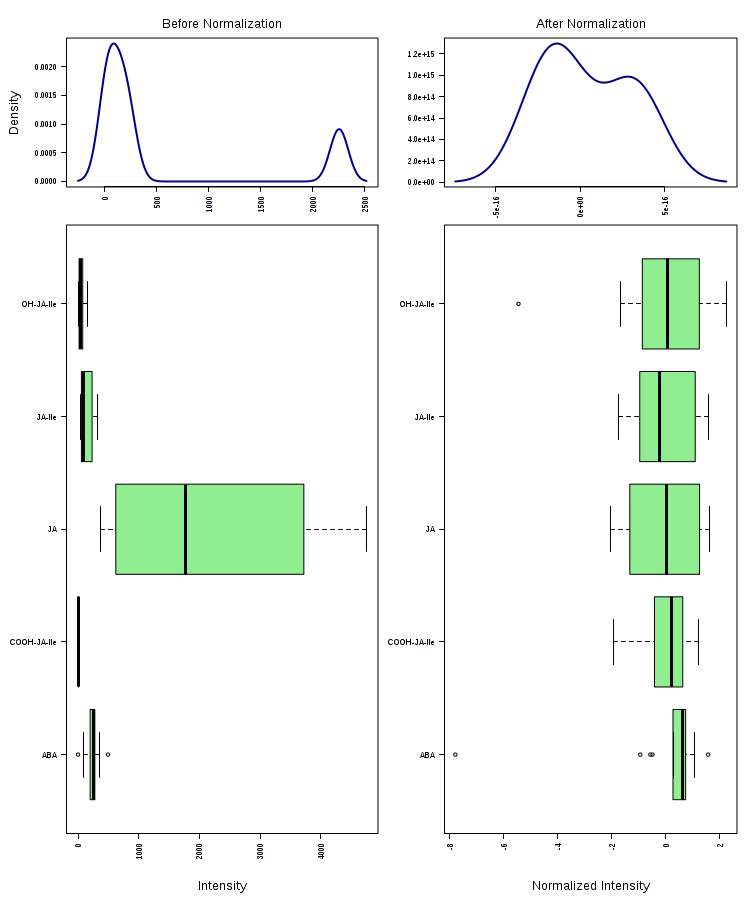

Supplement: Datasheet 1 — Source data files for the article. [file Data_Sheet_1.zip › Schuman2018_FPS_Source_Files/Metaboanalyst_W+OS_phytohormones/norm_2_dpi72.png]

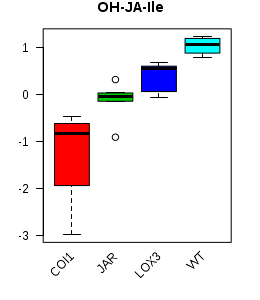

Supplement: Datasheet 1 — Source data files for the article. [file Data_Sheet_1.zip › Schuman2018_FPS_Source_Files/Metaboanalyst_W+OS_phytohormones/OH-JA-Ile_dpi72.png]

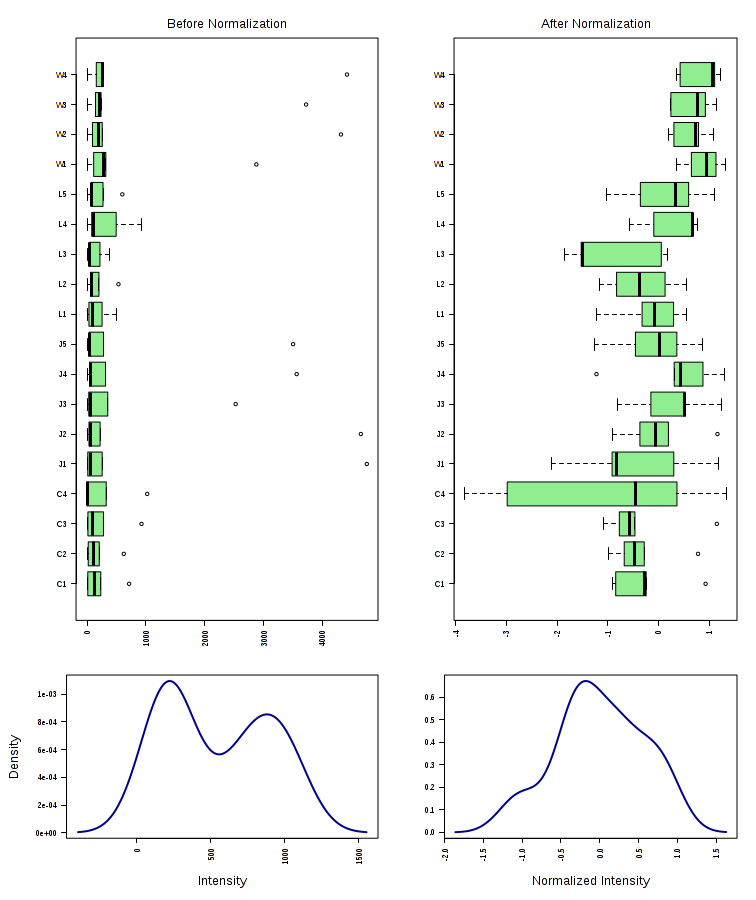

Supplement: Datasheet 1 — Source data files for the article. [file Data_Sheet_1.zip › Schuman2018_FPS_Source_Files/Metaboanalyst_W+OS_phytohormones/snorm_0_dpi72.png]

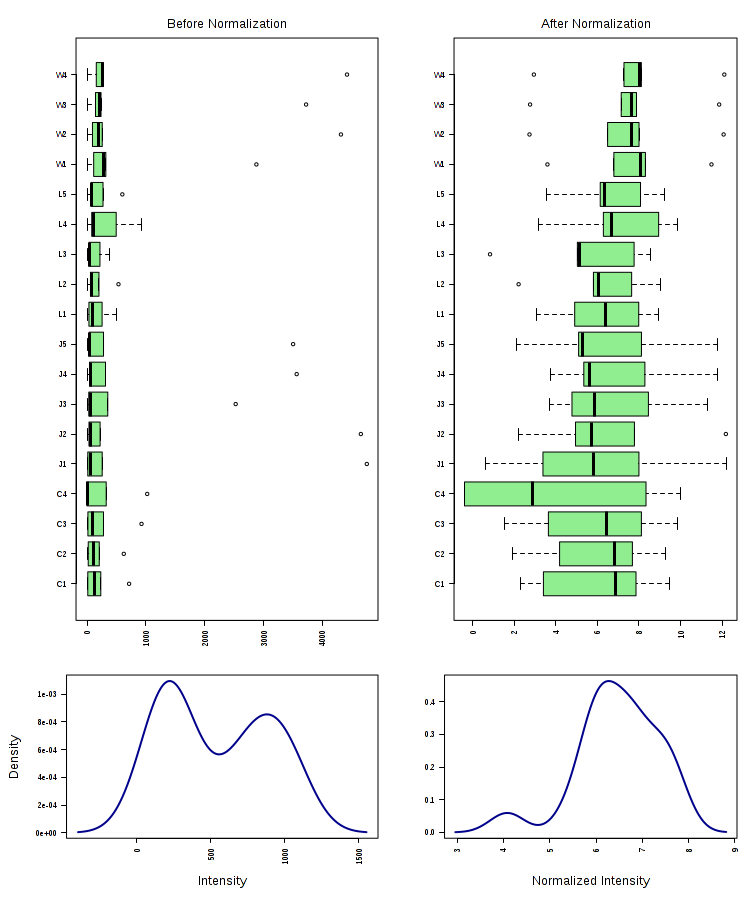

Supplement: Datasheet 1 — Source data files for the article. [file Data_Sheet_1.zip › Schuman2018_FPS_Source_Files/Metaboanalyst_W+OS_phytohormones/snorm_1_dpi72.png]

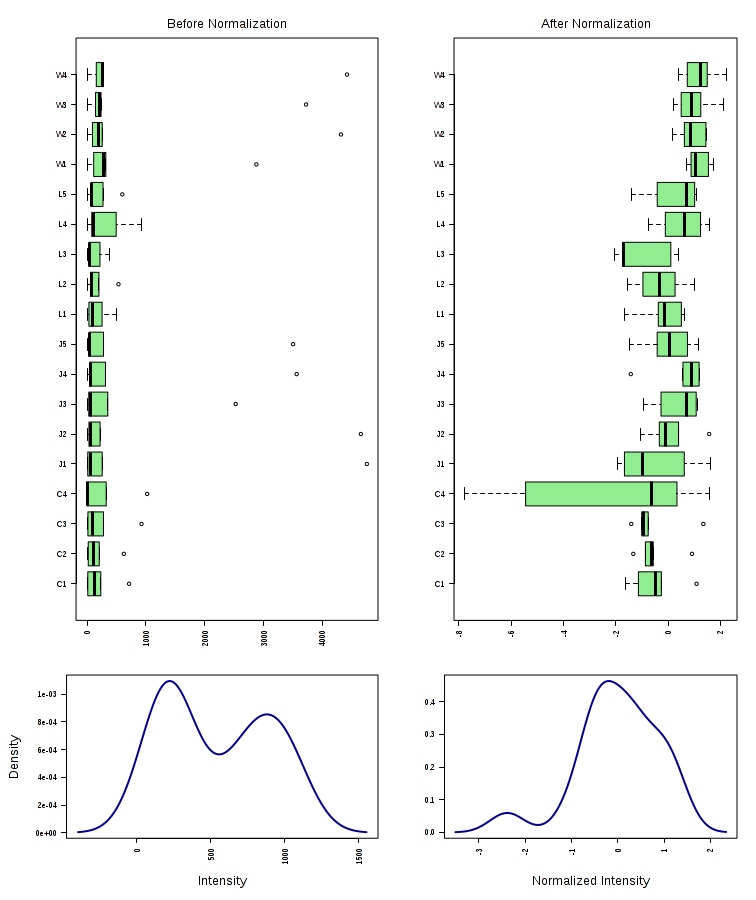

Supplement: Datasheet 1 — Source data files for the article. [file Data_Sheet_1.zip › Schuman2018_FPS_Source_Files/Metaboanalyst_W+OS_phytohormones/snorm_2_dpi72.png]
