# Supplementary material for: The Active Jasmonate JA-Ile Regulates a Specific Subset of Plant Jasmonate-Mediated Resistance to Herbivores in Nature
Source: Front Plant Sci. 2018 Jun 14;9:787. doi: 10.3389/fpls.2018.00787 (PMC6010948; doi:10.3389/fpls.2018.00787)
Supplement: Supplementary Figure 1 — JA and JA-Ile accumulation after mock herbivory treatment (W+OS) in WT, asLOX3, irCOI1, and irJAR4xirJAR6 plants (n = 3–5 plants); JA-Ile data for WT and irJAR4xirJAR6 plants is also shown in Figure 3B. a, bDifferent letters represent statistically significant differences (P < 0.0001) in Tukey's HSD post-hoc tests following one-way ANOVAs with a false discovery rate correction for multiple analytes, after log transformation and mean-centering to achieve normality and homogeneity of variance. See also Table 1 and Figure 3B. [file Image_1.pdf]

Supplementary Material

The active jasmonate JA-Ile regulates a specific subset of plant jasmonate-mediated resistance to herbivores in nature

Meredith C. Schuman*, Stefan Meldau, Emmanuel Gaquerel, Celia Diezel, Erica A. McGale, Sara Greenfield, and Ian T. Baldwin

*** Correspondence:** Meredith C. Schuman: mschuman@ice.mpg.de

1 Supplementary Data

All source data files are included as supplementary data (Schuman2018_FPS_Source_Files.zip).

2 Supplementary Figures

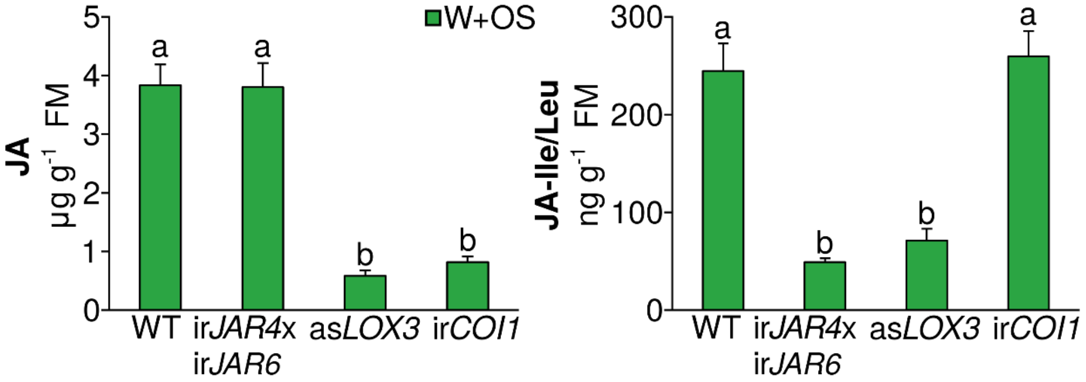

Supplementary Figure 1. JA and JA-Ile accumulation after mock herbivory treatment (W+OS) in WT, as*LOX3*, ir*COI1*, and ir*JAR4*xir*JAR6* plants (n = 3-5 plants); JA-Ile data for WT and ir*JAR4*xir*JAR6* plants is also shown in **Figure 3B**. [a,b]Different letters represent statistically significant differences (P<0.0001) in Tukey's HSD *post-hoc* tests following one-way ANOVAs with a false discovery rate correction for multiple analytes, after log transformation and mean-centering to achieve normality and homogeneity of variance. See also **Table 1** and **Figure 3B**.

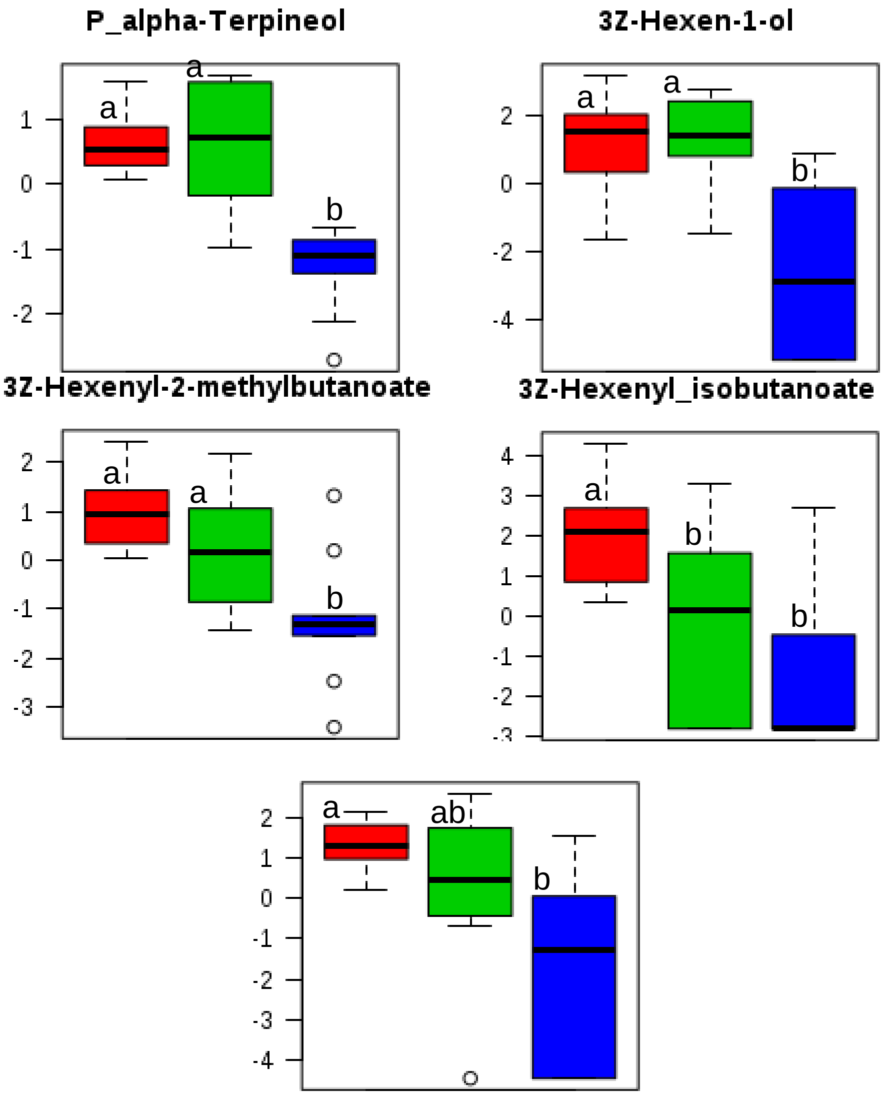

Supplementary Figure 2. Boxplots showing normalized peak areas of analytes which differed significantly by plant genotype (left to right: WT, red; ir*JAR4*x*irJAR6*, green; or as*LOX3*, blue) in headspace measurements of leaves on field-grown plants before W+OS treatment (control), to accompany **Table 2** (n = 10 plants). The lowest boxplot shows data from an unidentified green leaf volatile. [a,b]Different letters represent statistically significant differences in Tukey *post-hoc* tests following a one-way ANOVA and corrected for multiple testing using the false discovery rate method, after log transformation and mean-centering to achieve normality and homogeneity of variance.

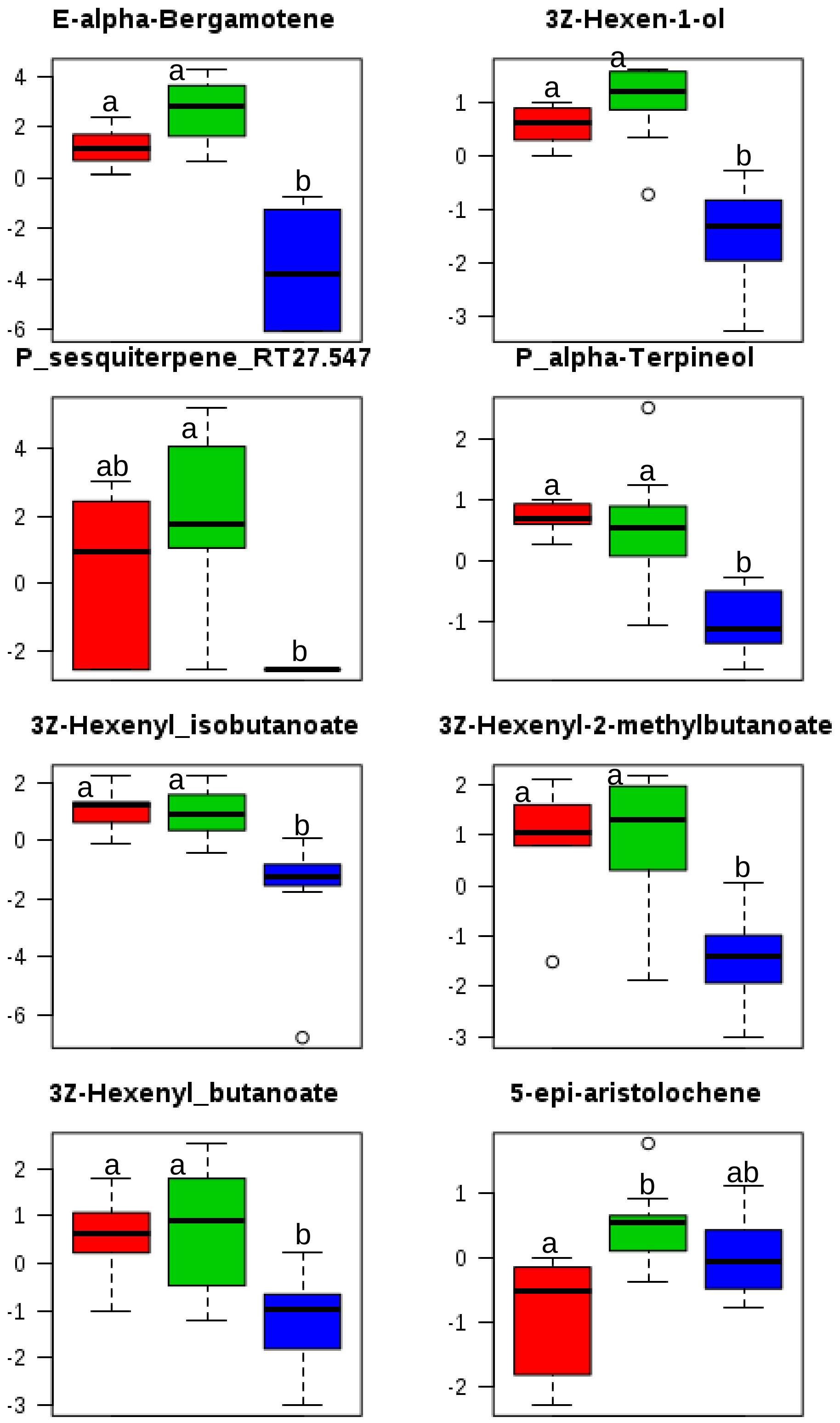

E-alpha-Bergamotene
4
2
0
-2
-4
-6
a
a
b
3Z-Hexen-1-ol
1
0
-1
-2
-3
a
a
b
P_sesquiterpene_RT27.547
4
2
0
-2
ab
a
b
P_alpha-Terpineol
2
1
0
-1
a
a
b
3Z-Hexenyl_isobutanoate
2
0
-2
-4
-6
a
a
b
3Z-Hexenyl-2-methylbutanoate
2
1
0
-1
-2
-3
a
a
b
3Z-Hexenyl_butanoate
2
1
0
-1
-2
-3
a
a
b
5-epi-aristolochene
1
0
-1
-2
a
b
ab

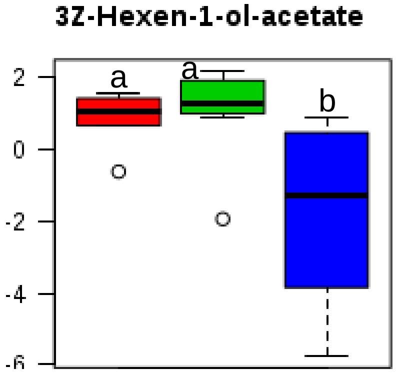

Supplementary Figure 3. Boxplots showing normalized peak areas of analytes which differed significantly by plant genotype (left to right: WT, red; ir*JAR4x*ir*JAR6*, green; or as*LOX3*, blue) in headspace measurements of W+OS-treated leaves on field-grown plants, to accompany **Table 2** (n = 5-8 plants). [a,b]Different letters represent statistically significant differences in Tukey *post-hoc* tests following a one-way ANOVA and corrected for multiple testing using the false discovery rate method, after log transformation and mean-centering to achieve normality and homogeneity of variance.

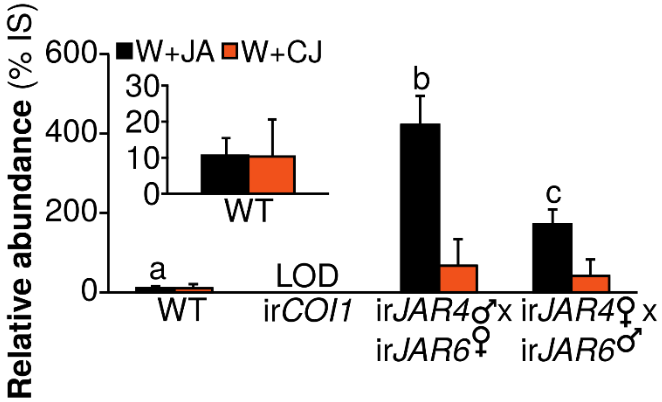

Supplementary Figure 4. A known jasmonate elicitor of volatiles, *cis*-jasmone, is not more potent than JA in eliciting (*E*)-α-bergamotene emission (n = 4-5 plants). Addition of either JA or an equimolar amount of *cis*-jasmone (CJ) affects (*E*)-α-bergamotene emission similarly in WT plants, but only JA and not *cis*-jasmone enhances emission in ir*JAR4*xir*JAR6* plants; results are shown separately for reciprocal crosses of the same ir*JAR4* and ir*JAR6* lines. [a,b]Different letters indicate significantly different emission of (*E*)-α-bergamotene (P<0.05 after a Holmes-Bonferroni *post-hoc* correction) in Mann-Whitney U-tests following a significant Kruskal-Wallis test across all genotypes.

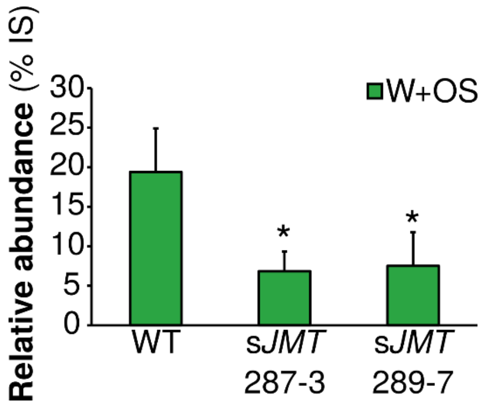

Supplementary Figure 5. Methyl jasmonate is not the elicitor of (*E*)-α-bergamotene. Two independently transformed transgenic lines ectopically expressing the *Arabidopsis thaliana* jasmonate methyltransferase (s*JMT*), which converts jasmonates to methyl jasmonate, have reduced emission of (*E*)-α-bergamotene from leaves following W+OS treatment (n = 9-10 plants). *s*JMT* lines differ significantly from WT (P<0.05 after a Holmes-Bonferroni *post-hoc* correction) in Mann-Whitney U-tests following a significant Kruskal-Wallis test across all genotypes.
